# Multi-Indel: A Microhaplotype Marker Can Be Typed Using Capillary Electrophoresis Platforms

**DOI:** 10.3389/fgene.2020.567082

**Published:** 2020-10-23

**Authors:** Shengqiu Qu, Meili Lv, Jiaming Xue, Jing Zhu, Li Wang, Hui Jian, Yuqing Liu, Ranran Zhang, Lagabaiyila Zha, Weibo Liang, Lin Zhang

**Affiliations:** ^1^Department of Forensic Genetics, West China School of Basic Medical Sciences and Forensic Medicine, Sichuan University, Chengdu, China; ^2^Department of Immunology, West China School of Basic Medical Sciences and Forensic Medicine, Sichuan University, Chengdu, China; ^3^Department of Forensic Medicine, Sichuan Police College, Luzhou, China; ^4^Department of Obstetrics and Gynecology, West China Second University Hospital, Sichuan University, Key Laboratory of Birth Defects and Related Diseases of Women and Children (Sichuan University), Ministry of Education, Chengdu, China; ^5^Department of Forensic Medicine, School of Basic Medical Sciences, Central South University, Changsha, China

**Keywords:** multi-indel, microhaplotype, capillary electrophoresis, forensic genetics, paternity tests

## Abstract

Since the concept of microhaplotypes was proposed by Kidd in 2013, various microhaplotype markers have been investigated for various forensic purposes, such as individual identification, deconvolution of DNA mixtures, or forensic ancestry inference. In our opinion, various compound markers are also regarded as generalized microhaplotypes, encompassing two or more variants in a short segment of DNA (e.g., 200 bp). That is, a set of variants (referred to herein as multi-variants) within a certain length includes single nucleotide polymorphisms (SNP), insertion/deletion polymorphisms (Indels), or short tandem repeat polymorphisms (STRs). At present, multi-variant is mainly aimed at multi-SNPs. However, the haplotype genotyping of multi-variants relies on single-strand analysis, mainly using massively parallel sequencing (MPS). Here, we describe a method based on a capillary electrophoresis (CE) platform that can directly obtain haplotypes of individuals. Several microhaplotypes consisting of three or more Indels with different insertion or deletion lengths in the range of less than 200 bp were screened out, each of which had at least three haplotypes. As a result, the haplotype of an individual was reflected by the length of its polymorphism. Finally, we established a multiplex amplification system containing 18 multi-Indel markers that could identify haplotypes on each chromosome of an individual. The combined power of discrimination (CPD) and the cumulative probability of exclusion (CPE) were 0.999999999997234 and 0.9984, respectively.

## Introduction

Owing to various forensic cases encountered in practice, compound markers have attracted the interest of forensic DNA scientists. Compound biomarkers consisting of two or more variants that occur in short DNA segments of ∼200 bp for example, can be regarded as generalized microhaplotypes, including insertion and deletion polymorphisms (Indels) closely linked to short tandem repeat polymorphisms (STRs) (DIP-STR), single nucleotide polymorphisms (SNP) closely linked to STR (SNP-STR), Indel polymorphisms closely linked to SNP (DIP-SNP), and several Indel polymorphisms linked very tightly in physical positions (multi-Indels) (Castella et al., 2013; Wang et al., 2015; Wendt et al., 2016; Tan et al., 2017; Tan et al., 2018; Oldoni and Podini, 2019).

Haplotypes are presently interpreted in three ways. A statistical inference method was used after separately genotyping each locus, but it could not reflect the true haplotype of individuals (such as PHASE) (Kong et al., 2008; Kidd et al., 2013, 2014). Other ways to interpret include the use of DIP-STR, SNP-STR, DIP-SNP, SNP-SNP, or other compound markers for detection. By designing allele-specific PCR primers, the 3′ end of a PCR primer is paired with upstream DIP or SNP alleles. A shared reverse primer is then designed downstream of other STR or SNP markers. Thereafter, two allele-specific sequences are obtained using PCR. The genotype of haplotype markers from an individual can be determined using a capillary electrophoresis (CE) platform and a two-step detection method, but the phase of a haplotype can be unambiguously determined only when the microhaplotypes include two variants (Castella et al., 2013; Cereda et al., 2014; Oldoni et al., 2015; Tan et al., 2017; Liu et al., 2018, 2019; Moriot and Hall, 2019; Oldoni and Podini, 2019; Zhang et al., 2020). Additionally, the main limitation of microhaplotype markers comprising only two variants is the difficulty with increasing polymorphism. A third method relies on single-stranded haplotypes that are resolved by experimental analyzes such as massively parallel sequencing (MPS), which can directly detect the phases of haplotypes on sequenced strands (Borsting and Morling, 2015; Snyder et al., 2015; Wang et al., 2015; Wendt et al., 2016; Chen et al., 2019; Turchi et al., 2019; Zhu et al., 2019; de la Puente et al., 2020; Pang et al., 2020; Sun et al., 2020). However, forensic scientists face many practical challenges due to the complexity of MPS, extensive data processing requirements, and higher costs.

Since the discovery and identification of 2,000 human diallelic Indels in 2002, many studies have found that Indels can serve as important complements to forensic genetic markers in addition to STR and SNP (Weber et al., 2002). Compared to STR, Indel amplicon fragments are shorter, and mutation rates are lower. Compared to SNP, Indels have length polymorphism, which can be directly detected by CE of PCR products. This can be easily achieved in most forensic DNA laboratories without complex detection methods. However, most Indels have only two alleles, the polymorphisms are relatively poor and the discriminatory power is relatively lower than that of STR. The present study considered a marker containing at least two Indel loci in a short segment of DNA (namely multi-Indel), as a new microhaplotype. This marker not only increased Indel polymorphism, but also retained the advantages of SNP and STR. Since Indels are markers with length polymorphism, we selected Indel loci with different allele lengths to form a microhaplotype that was directly detectable by CE. According to length polymorphism, it unambiguously reflected the phases of haplotypes from individuals.

Previous studies of multi-Indels have been limited to increasing polymorphism. However, as the length of an insertion or deletion in alleles of an Indel is not specific, some polymorphism information is lost (Huang et al., 2014; Sun et al., 2016). Additionally, individual haplotypes have been statistically inferred after genotyping each Indel locus, which does not reflect the true haplotype of an individual (Zhao et al., 2018). In the present study, we proposed a strategy based on a CE platform to obtain accurate haplotypes of individuals, and constructed a multiplex amplification system containing 18 multi-Indel markers to improve the discrimination power of Indels.

## Materials and Methods

### Ethics

The participants provided their written informed consent to participate in this study and for participants under the age of 16, the legal guardian provided written informed consent to participate. All samples were obtained under the supervision of the Ethical Committee of the Sichuan University (KS2019042).

### Samples and DNA Extraction

This study included 335 samples of EDTA blood collected from the Sichuan Province, China. The samples were collected under written informed consent from 170 unrelated Sichuan Han individuals, 30 unrelated Sichuan Yi individuals, and 83 parent–child pairs. Notably, 134 samples were from 17 unrelated extended families that descended from 83 parent–child pairs; thus, some parent–child pairs had the same alleged parent or alleged child. We extracted DNA using the BioTeke DNA kits (BioTeke Corp., Beijing, China) as described by the manufacturer. The collected DNA was quantified using the NanoDrop^TM^ 1000 spectrophotometer (Thermo Fisher Scientific Inc., Waltham, MA, United States).

### Selection of Multi-Indel Markers

Candidate Indels were selected from 208 samples including 103 Han Chinese in Beijing, China (CHB) and 105 Southern Han Chinese in China (CHS) in the 1000 Genomes Project phase 3 using VCFtools^1^ (Sudmant et al., 2015) that met the following criteria: being biallelic, minor allele frequency (MAF) >0.1, located in a non-coding region or intron, allele length of each Indel ranged from 1 to 30 bp; one multi-Indel comprised at least three Indels, physical distance between selected Indels in one multi-Indel marker was <200 bp, alleles had different lengths, and the length of any allele was not equal to the sum of the lengths of the other two or more alleles (each theoretical haplotype has a unique amplicon length), different multi-Indel markers were >10 Mb apart if on the same chromosomal arm, no other Indel variation had MAF >0.005 within this range, the haplotype frequency calculated by Haploview was ≥3, and at least three haplotypes had a frequency of ≥0.05 (Barrett et al., 2005).

### Genotyping Multi-Indels

#### Primer Design and Optimization

We designed PCR primers using the online tool Primer3web^2^ according to the following criteria: PCR product size, 70–250 bp; Tm values, 55–62°C, and GC content, 30–60%. Potential secondary structures between obtained primer pairs (including formation of primer dimers and hairpin structures, were examined using AutoDimer^3^, and specific primers were identified using Primer-BLAST^4^. All primer pairs were then assigned according to the predicted amplicon length, and one of the primer pairs was labeled with the fluorochromes, FAM, HEX, TAMRA, and ROX. All primers were synthesized (Thermo Fisher Scientific Inc.) then purified using high performance liquid chromatography (HPLC). Subsequently, we used 1–5 samples to perform a singleplex PCR reaction for each microhaplotype locus. CE was used to detect the PCR products of each microhaplotype locus. And the homozygous samples were amplified using the corresponding primers that are not labeled with fluorescent dyes for Sanger sequencing verification. The size of each locus was examined and compared with the size of CE to determine the electrophoretic mobility of each allele.

#### Multiplex PCR Amplification

In multiplex RCR amplification, the initial each primer concentration was 0.2 μM. Then this multiplex amplification system was then optimized based on primer concentrations and peak heights. We programmed the thermal cycler according to the manufacturer’s instructions. In order to minimize the influence of the annealing temperature of the multiplex system, 18 multi-Indel markers were multiplex amplified under different annealing temperature gradients (56.9, 57.6, 58.4, and 59.1°C) and different PCR cycles (25, 27, 29 and 32) with 1 ng of control DNA F312. According to the optimized and relatively balanced genotyping profiles, the optimal annealing temperature and optimal cycle number of our system were finally determined. The final reaction volume of 10 μL included 5 μL of 2× Multiplex PCR Master Mix (Qiagen GmbH, Hilden, Germany), 2 μL of primer mixture, 1 μL of target DNA (1 ng/μL), and 2 μL of RNase-free water. The samples were amplified by PCR using the GeneAmp 9700 PCR System (Applied Biosystems, Foster City, CA, United States) under the following cycle conditions: 95°C for 15 min, then 27 cycles of 30 s at 94°C, 90 s at 58.4°C, 60 s at 72°C, and hold at 60°C for 60 min. All 335 samples were genotyped using the 18 multi-Indel markers in one multiplex PCR reaction.

#### Detection and Analysis

The PCR products were detected using the ABI 3500 Genetic Analyzer (Applied Biosystems) and a preloaded AGCU E5 dye fragment analysis run module. Samples were prepared for CE by mixing 1 μL of the PCR products with 8.9 μL of Hi-Di formamide (Applied Biosystems) and 0.1 μL of SIZ500 size standard (AGCU ScienTech, Jiangsu, China). Samples were injected at 1.2 kV for 5 s and resolved by electrophoresis at 15 kV for 1,310 s in Performance Optimized Polymer-4 (POP-4 polymer) (Applied Biosystems). Genotyping data were then analyzed using the GeneMapper^TM^ ID Software v3.2.1 (Applied Biosystems), with an allele peak threshold of 100 relative fluorescence units (RFU).

### Allele Nomenclature

Since a nomenclature system for multi-Indel markers has not been standardized and they are essentially a type of microhaplotype, we named the multi-Indel markers in this study according to those suggested by Kidd (2016). We labeled the smallest of their alleles as 0 according to the size of the amplicon in each multi-Indel marker, and if other alleles were N bp larger than the smallest allele, these were called N. New alleles identified in this study were also named according to their length (Huang et al., 2014).

### Sensitivity Study

We evaluated the sensitivity of our multiplex system. Serially diluted control DNA F312 (2 ng μL^–1^ stock) (Beijing Microread Genetics, Beijing, China) was amplified in triplicate with quantities of 1, 0.5, 0.25, 0.125, and 0.0625 ng. These samples were processed under the same reaction conditions described above.

### Mixture Studies

We assessed the ability of our multiple system to detect DNA in mixtures of several ratios of female and male DNA. Mixtures of female/male DNA samples (control DNA F312 and M308, Beijing Microread Genetics, Beijing, China) in ratios of 19:1, 9:1, 4:1, 3:1, 1:1, 1:3, 1:4, 1:9, and 1:19 ng were amplified in our multiplex assay in triplicate.

### Degradation Study

We simulated several degraded samples that were amplified and resolved by electrophoresis as described above to evaluate the ability of our multiple system to detect DNA in degraded samples. The control DNA M308 was ultrasonically degraded by 0, 100, 200, 300, or 400 cycles of 200 W for 10 s per cycle with 4-s intervals between cycles. The extracted DNA from the EDTA blood was ultrasonically degraded by 0, 200, 400, and 600 cycles of 400 W for 10 s per cycle, with 4-s intervals between cycles.

### Statistical Analysis

Each allele was considered as one haplotype. The allele frequency was the available haplotype frequency. The forensic parameters allele frequencies, power of discrimination (PD), power of exclusion (PE), typical paternity index (TPI), and observed heterozygosity (Ho), and the exact tests of the Hardy–Weinberg equilibrium (HWE) were calculated using a modified spreadsheet within PowerStat v1.2 (Promega Corp., Madison, WI, United States) (Zhao et al., 2003). Linkage disequilibrium (LD) in pairwise loci were analyzed using GENEPOP (Rousset, 2008). The effective number of alleles (A_*e*_) was calculated based on the formula proposed by Kidd and Speed (2015).

The paternity index (PI) is the likelihood ratio of the probability that an alleged father with the DNA result is the biological father of the child and the probability that the random man is the biological father of the child. The PI was calculated based on LR principles according to the International Society for Forensic Genetics (ISFG) (Gjertson et al., 2007). The combined paternity index (CPI) was equivalent to the product of PI for all multi-Indel markers tested in each parent–child pair.

## Results

### Marker Selection and General Information

We screened candidate Indels that met the inclusion criteria from the 1000 Genomes Project database. The filter of biallelic Indels with MAF > 0.1 and the allele length variation of each Indel from 1 to 30 bp resulted in 629,402 candidates, which were then filtered according to differences between allele lengths of all loci within a physical distance of <200 bp, and 26,092 potential haplotype markers remained. These were filtered according to each haplotype containing at least 3 Indels, which left 1,642 candidates. Loci in gene coding regions and those positioned <10 Mb apart on the same chromosomal arm were excluded. According to the number and the frequency of haplotypes calculated by Haploview and filtering according to our primer design criteria, only 52 candidates remained. Finally, 18 candidate multi-Indel markers containing 54 Indel loci were genotyped in one multiplex panel after removing loci for which correct genotype results could not be obtained due to long homopolymer structures or 2–15 nucleotide tandem repeats. Table 1 shows the general information of the 18 multi-Indel markers, and Supplementary Table S1 shows the haplotype frequency calculated by Haploview.

**TABLE 1:** The general information of 18 multi-Indel markers.

Microhaplotype	GRCh37	rs-Number dbSNP	Extent in bp	Allele1/Allele2	Insertion allele length	Primer sequences (label)	Primer concentration (μM)	Theoretical amplicon size (bp)
mh01zl001	2029533	rs368828322	16	A/AG	1	GGCGGGGTGAATAGTTTGAC (ROX)	1.569	150, 151, 160, 161, 179, 180, 170, 169
	2029539	rs372567620		A/AAGGTCAGAGC	10	TCAGTAAACAACCCCTGCCT		
	2029549	rs148361309		C/CAGGTGACCAGGAGTGACTA	19			
mh01zl002	100194878	rs55796544	25	C/CCT	2	TGTGCTCCTCTTTCTCACTAGT (TAMRA)	0.392	106, 107, 109, 110, 103, 104, 105, 108
	100194896	rs67810269		C/CTGTA	4	TTAAGATGGTCAGGGCATCAG		
	100194903	rs71075445		C/CT	1			
mh02zl001	30981778	rs142363578	142	C/CTTCT	4	CCCTTACTCCCTCTCGTCTTC (TAMRA)	0.196	196, 198, 200, 202, 215, 217, 213, 211
	30981829	rs144117237		T/TTC	2	GGAGGGATGAAGGGAGGC		
	30981920	rs148016741		C/CCCTCCCTCCCTCCCT	15			
mh02zl003	212161558	rs575990766	85	A/ACATATGTATG	10	ACTAAAGCCTGTATATGTAGCCT (ROX)	1.569	216, 232, 238, 242, 212, 222, 226, 228
	212161604	rs141442566		A/ATACATATGTATGTATG	16	CCCAGTATCATTCTCTATCTCTGC		
	212161643	rs66617012		A/ATAAG	4			
mh03zl001	73878996	rs34404453	47	T/TC	1	TGATTCTTCCTTACTCCTCCAAAG (HEX)	0.196	124, 125, 126, 129, 120, 121, 123, 128
	73879030	rs149171688		A/AAATAT	5	GGCAACAGAATAAGACTCCGTT		
	73879043	rs34483288		A/AATT	3			
mh03zl002	87352688	rs200679094	48	G/GAAATCTAAATAT	12	ACCATCTACATTTTCCCTGTAAA	0.588	118, 123, 130, 131, 119, 122, 134, 135
	87352693	rs370444413		A/AGGTG	4	GCTGGGTCATCGCCATTTT (TAMRA)		
	87352736	rs74604190		T/TA	1			
mh03zl003	163670527	rs80013016	102	T/TG	1	AGCTAGAGGTGTGTAGGCAA (FAM)	0.196	183, 184, 194, 195, 199, 210, 211, 200
	163670601	rs372207681		C/CAGGTGCCAGCT	11	GCTTCTGCGTGACACTGC		
	163670629	rs111507567		G/GCTGCTGCTTTGGGCAA	16			
mh04zl001	18391312	rs11282557	17	G/GACAGTATTT	9	CCTTGTTGCTGCAGTAGAAAAT (ROX)	0.147	135, 144, 146, 155, 158, 147, 156, 167
	18391316	rs58595156		G/GGAAAAATTGCT	11	TGATCACTTAAGTTCGATGAAAGAA		
	18391329	rs372089291		A/ATTCTCCTAAATT	12			
mh04zl003	187124231	rs66502037	82	C/CAT	2	TGCGCATATACACATACATAGATG	0.098	150, 153, 155, 157, 151,146, 152, 148
	187124238	rs77222977		G/GCACA	4	ATGTGTATGGGGTTGTGCAC (TAMRA)		
	187124313	rs71871946		T/TTCATA	5			
mh07zl001	57322877	rs71053237	103	C/CTAAATGAT	8	TTGTGGGGTGGCGGAAG	0.392	172, 179, 180, 182, 169, 171, 174, 177
	57322974	rs72447238		T/TATA	3	GCACTGGATGGCACTCTTTT (HEX)		
	57322980	rs71053238		A/AAT	2			
mh10zl001	7140235	rs145059123	72	C/CA	1	ACACATTCACACATTCATTTAGACA (ROX)	1.569	215, 216, 218, 221, 224, 217, 222, 223
	7140259	rs539040996		T/TAGACAC	6	TGGTGTGTGTGTATGCTAGTG		
	7140307	rs34860860		T/TCA	2			
mh10zl002	113582580	rs143378119	38	G/GAGAATACATTA	11	GAACAGAGTGTCATCCATTTTCT	0.098	110, 112, 115, 121, 126, 137, 143, 148
	113582616	rs370632025		T/TTATGG	5	TCAGGCCAATCACACGTG (FAM)		
	113582618	rs571358799		C/CAGGACTGGAAGGAGAATACAAT	22			
mh13zl001	25442007	rs58303500	26	G/GCAT	3	GCCGTGATCTTCCTGGGAA (FAM)	0.196	217, 218, 220, 224, 214, 215, 221, 223
	25442012	rs34156563		A/AT	1	TAATGAGGGCTGGGGTGTTT		
	25442033	rs67523118		A/ACTTATT	6			
mh18zl001	61672654	rs377195018	16	A/AGAGGTGGGACC	11	GAATGCCGTCTTCCACCAAA (FAM)	0.196	147, 153, 162, 164, 149, 151, 158, 160
	61672667	rs59925455		T/TTGGG	4	AGGGGCAAGGTAGTTCTCTG		
	61672670	rs201823781		G/GAT	2			
mh19zl001	490362	rs138906215	87	A/AAAAG	4	GGCGACAAGAGTGAAACTC	0.784	155, 157, 159, 162, 153, 160, 164, 166
	490414	rs35679623		A/ACT	2	CTCAGCGTGAACAAAGAGTG (HEX)		
	490449	rs11283323		C/CAATACTG	7			
mh19zl002	56039183	rs543507020	85	A/ATGCACACACTCACAATTGCACACACG	26	TGGACACACGCACACTGG (FAM)	0.588	177, 181, 201 203, 175, 179, 205, 207
	56039213	rs559033587		A/ACACT	4	TCTGTGCAAGTGTGAATGTCG		
	56039268	rs36127315		G/GCA	2			
mh21zl001	21261329	rs562677243	5	T/TTA	2	AGCAATGTGTTCACAGATACCA	0.686	164, 167, 174, 176, 165, 166, 173, 175
	21261333	rs576365249		C/CG	1	AGGCCATGGAGAGGAGTAGA (TAMRA)		
	21261334	rs373980639		G/GGGGACATTT	9			
mh21zl002	42384738	rs147144385	55	T/TA	1	ACACATTCTCAAGCACTCACA (HEX)	0.049	147, 149, 150, 151, 144, 145, 146, 148
	42384760	rs200218606		T/TCACA	4	GGTGGGAGATGTGAATGTGT		
	42384793	rs138205093		A/ACT	2			

### Multiplex Assays

**FIGURE 1:**
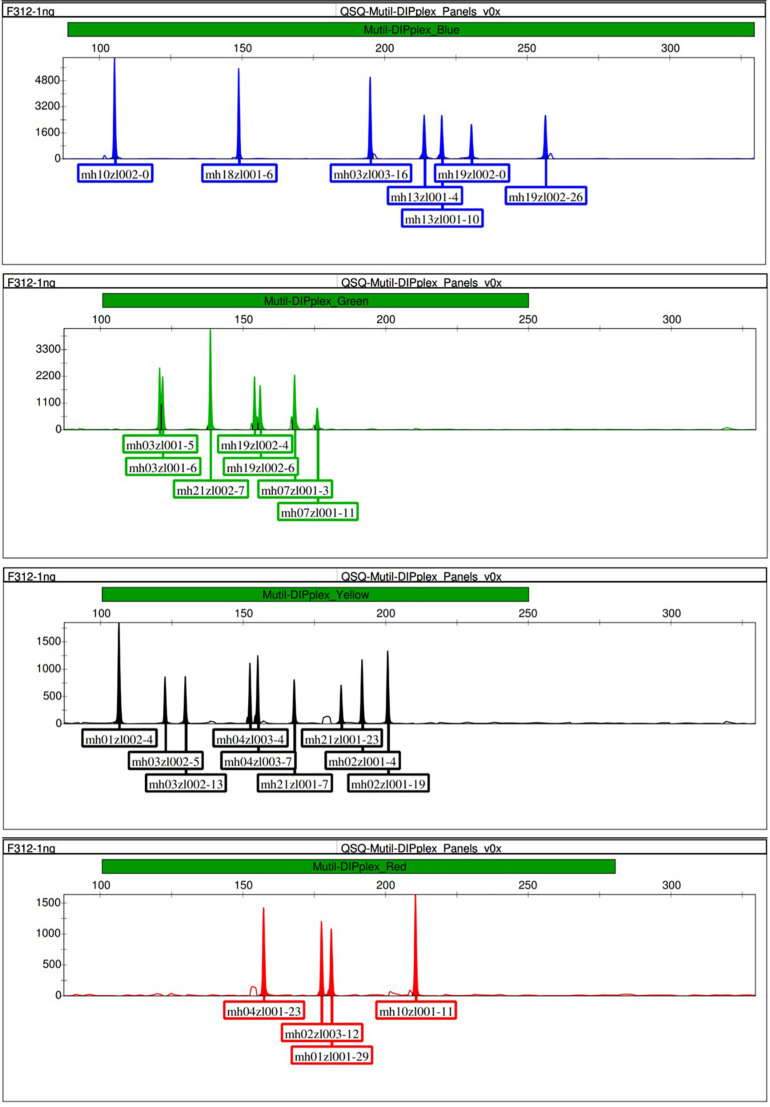
Representative electropherogram of control DNA F312 amplified at 1 ng.

Before performing the multiplex amplification, we verified the amplification of the primer pairs at each marker by performing singleplex PCR reaction and detection by CE. The size of the allele was determined based on the results of Sanger sequencing of the corresponding homozygous samples. The CE detection results of the singleplex PCR reaction and the Sanger sequencing of the corresponding markers were shown in the Supplementary Data. After the development and optimization of this multiplex panel, 18 microhaplotype markers were successfully amplified in a single PCR reaction, and the optimal temperature was determined as 58.4°C, the optimal cycle number was determined as 27, following the optimized PCR conditions presented in section “Multiplex PCR Amplification.” After one PCR reaction and the next CE run, 18 multi-Indel markers containing 54 Indel loci were genotyped per DNA sample. The results showed that 18 complete profiles were detected in each test sample. Figure 1 shows an example of capillary electropherogram obtained by genotyping the control DNA F312. Supplementary Figure S1 shows a capillary electropherogram of the control DNA M308, and Table 1 includes information about the sequences and concentrations of all primers in the system.

### Sensitivity Study

**FIGURE 2:**
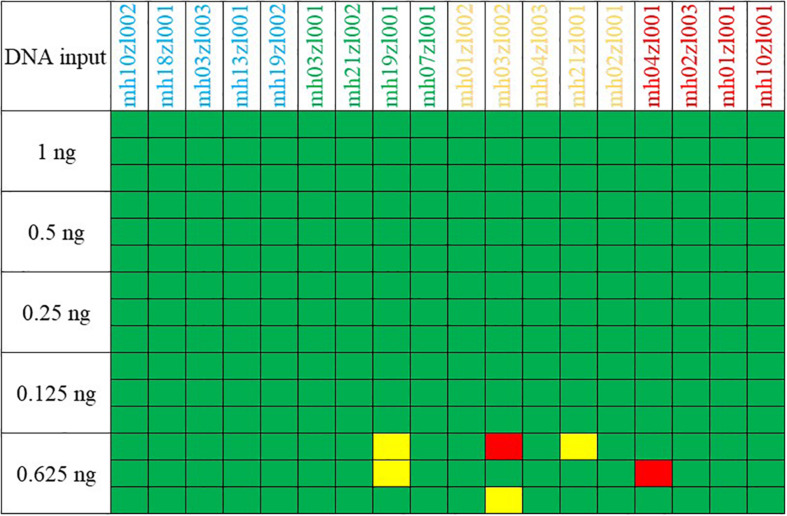
Results of a sensitivity study using serially diluted control DNA F312. Green boxes, no allele drop-out; red boxes, no alleles recovered; yellow boxes, only one of two expected heterozygote alleles was called.

The sensitivity of our multiplex assay was tested with control DNA F312 serially diluted to template amounts of 1, 0.5, 0.25, 0.125, and 0.0625 ng. Each template amount was amplified three times. Sample inputs >0.125 ng consistently generated full profiles (Figure 2) when amplified for 27 PCR cycles and when the threshold for allele calls was 100 RFU. As the template DNA concentration was gradually reduced from 1 to 0.125 ng, the average detected peak height shifted from 4,144 to 351 RFU. When the template DNA F312 decreased to 0.0625 ng, profiles were partial and an average 91.36% of the allele was detected with an average peak height of 212 RFU. Therefore, our multiplex system obtained reliable profiles at a threshold of 100 RFU above a DNA concentration of 0.125 ng.

### Mixture Studies

**FIGURE 3:**
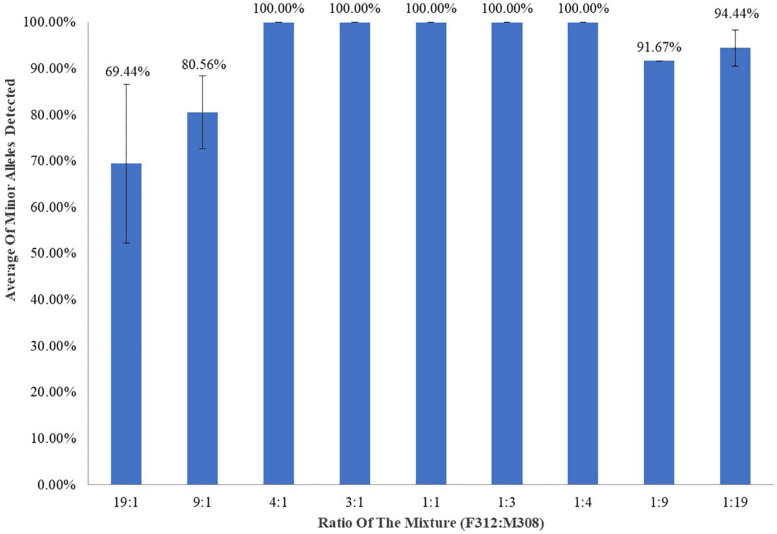
F312/M308 DNA amplification using 1 ng of total DNA and assessed in triplicate.

Template DNA (1 ng) comprising a mixture of control DNA F312 and 19:1, 9:1, 4:1, 3:1, 1:1, 1:3, 1:4, 1:9, and 1:19 ratios of M308 was tested in triplicate. All unique minor profiles were called at ratios of 4:1, 3:1, 1:1, 1:3, and 1:4 (Figure 3), and minor alleles were called at averages of 80.56% and 91.67% at ratios of 9:1 and 1:9, respectively. The unique minor profile in the mixture was called at averages of 69.44 and 94.44% at ratios of 19:1 and 1:19, respectively.

### Degradation Study

**FIGURE 4:**
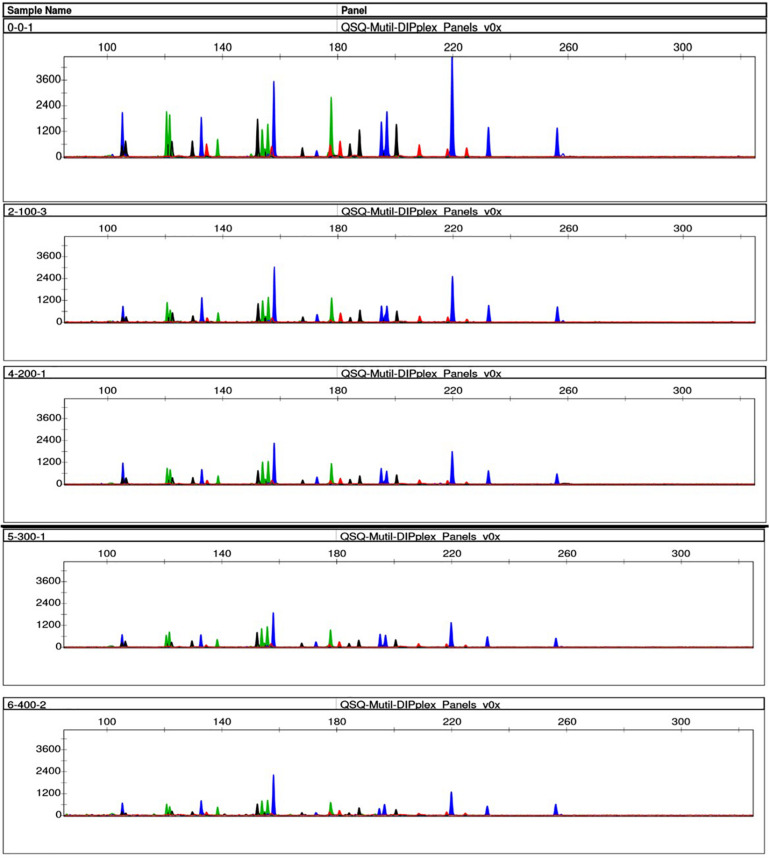
Effects of ultrasound on degraded control DNA M308. We degraded control DNA using 0 **(top)**, 100, 200, 300, and 400 **(bottom)** ultrasonic cycles of 200 W for 10 s per cycle, with 4 s between cycles.

**FIGURE 5:**
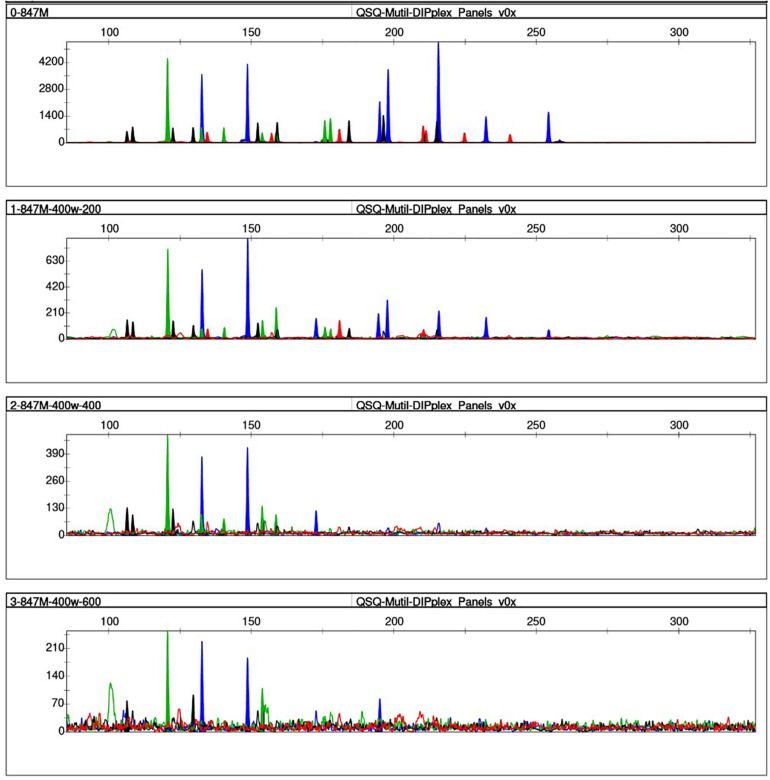
Effects of ultrasound on DNA extracted from fresh EDTA blood. We degraded DNA using 0 **(top)**, 100, 200, 300, and 400 **(bottom)** ultrasonic cycles of 200 W for 10 s per cycle, with 4 s between cycles.

We simulated the degradation of the control DNA M308, and DNA extracted from fresh EDTA blood to determine the effects of sample degradation. After the control DNA M308 was disrupted using 0–400 ultrasound cycles of 200 W, full profiles were obtained using a peak height analysis threshold of 100 RFU. However, the average peak height gradually decreased as the number of cycles increased (Figure 4). Only 83% of the alleles were called from the DNA sample extracted from fresh EDTA blood (a conventional case sample), after 200 ultrasound cycles at 400 W, and after 400 and 600 cycles, 33.33 and 23.33% of alleles were called, respectively (Figure 5).

### Statistical Analysis

**FIGURE 6:**
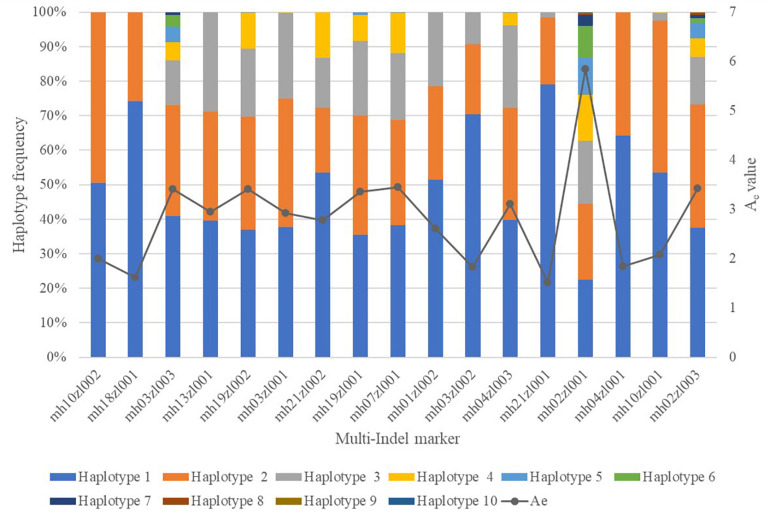
Effective numbers of alleles (A_*e*_) and haplotype frequencies of 17 multi-Indel markers.

We genotyped 200 unrelated individuals from Sichuan using our panel of 18 multi-Indel markers containing 54 Indel loci multiplex systems. Supplementary Table S2 shows their genotype profiles. The mean distance between the outermost Indels of each multi-Indel was 58 (5–142) bp. The average amplicon length was 182 (107–326) bp. The actual and theoretical amplicon sizes differed in seven multi-Indel markers. Our multiplex detected 77 specific amplicons (that is, 77 haplotypes) in 200 Sichuan individuals. One of these, mh01zl001, was monomorphic in the surveyed population, so we excluded this locus from further statistical analysis. We found 2, 3, 4, 5, 7, 9, and 10 haplotypes in 3, 4, 3, 4, 1, 1, and 1 multi-Indel markers respectively. Supplementary Table S3 lists the alleles of 17 multi-Indel markers and their frequencies. The mean and median values of A_*e*_ for these 17 loci were 2.83 and 2.92, respectively (Figure 6).

We also tested each locus for conformity to the HWE model and for potential LD. The threshold *p* value for the HWE test was set at 0.00037 after the Bonferroni correction, and no deviations from linkage equilibrium were significant between pairwise loci after the Bonferroni correction (*p* > 3.68 × 10^–4^; Supplementary Table S4). Table 2 lists the PD, PE, Ho, PM, PIC, TPI, and *p* values for HWE of the 17 multi-Indel markers. The average PD value was 0.7585 (range, 0.5146–0.9469). The average PE value for the 17 loci was 0.591 (range, 0.0888–0.5535). The Ho was 0.355 to 0.775, and combined PD and combined PE were 0.999999999997234 and 0.998414249965817, respectively.

**TABLE 2:** Values for PD, PE, Ho, PM, PIC, TPI, and HWE of a 17 multi-Indel marker.

Microhaplotype	PD	PE	Ho	PM	PIC	TPI	HWE (*p*)
mh01zl002	0.78135	0.315571	0.62	0.21865	0.545721	1.315789	0.934909564
mh02zl001	0.9469	0.553495	0.775	0.0531	0.806189	2.222222	0.034566771
mh02zl003	0.8668	0.36213	0.655	0.1332	0.658147	1.449275	0.092087668
mh03zl001	0.7905	0.459875	0.72	0.2095	0.583465	1.785714	0.069324435
mh03zl002	0.64445	0.174709	0.485	0.35555	0.403443	0.970874	0.387110874
mh03zl003	0.8471	0.36213	0.655	0.1529	0.659459	1.449275	0.09720049
mh04zl001	0.60385	0.151068	0.455	0.39615	0.353869	0.917431	0.875124703
mh04zl003	0.83395	0.369131	0.66	0.16605	0.612997	1.470588	0.560030079
mh07zl001	0.8645	0.436037	0.705	0.1355	0.657207	1.694915	0.837279591
mh10zl001	0.6726	0.174709	0.485	0.3274	0.408344	0.970874	0.308612984
mh10zl002	0.6298	0.178899	0.49	0.3702	0.374975	0.980392	0.751342383
mh13zl001	0.8158	0.315571	0.62	0.1842	0.586598	1.315789	0.20736369
mh18zl001	0.54335	0.110909	0.395	0.45665	0.309277	0.826446	0.734621995
mh19zl001	0.84985	0.42826	0.7	0.15015	0.645455	1.666667	0.908230714
mh19zl002	0.8661	0.383394	0.67	0.1339	0.651957	1.515152	0.236016462
mh21zl001	0.5146	0.0888	0.355	0.4854	0.289889	0.775194	0.621845819
mh21zl002	0.8229	0.284923	0.595	0.1771	0.594375	1.234568	0.168954084

### Application in Paternity Testing

We analyzed 83 parent–child pairs and calculated PI using genotype data using the multi-Indel multiplex panel. Supplementary Table S5 shows the genotypes of 83 parent–child pairs and the specific PI per locus and CPI per parent–child pair. The allele frequency of 17 multi-Indel markers was obtained separately from the 200 unrelated individuals. All the parent–child pairs conformed to the Mendelian laws of inheritance. No mutation or recombination was found in any of the multi-Indel markers from 83 parent–child pairs. Overall, the CPI in 83 parent–child pairs determined by the panel of 17 multi-Indel markers averaged 2.82066955485148 × 10^6^ (range, 0.58394420522483 × 10^3^ to 5.06111014257473 × 10^7^. Fourteen parent–child pairs had a CPI below 10,000, which did not support a biological parent–child relationship between them. However, their CPI were >0.0001, so a biological parent–child relationship cannot be excluded. The number of loci would need to be increased, or combined with STR kits to clarify this situation.

## Discussion

Multi-variant is slightly different from the traditional microhaplotype. We believe that a set of all variants including SNP, Indel and STR within a specifically short length can be considered as generalized microhaplotypes. Only microhaplotypes containing two SNP can presently be genotyped on the CE platform due to limitations of the system (Zhang et al., 2020). Therefore, we selected Indels from the 1000 Genomes Project as the basis for constructing microhaplotypes that could be analyzed using this platform. The human Indel mutation rate ranges from 0.53 to 1.5 × 10^–9^ per base per generation (Kondrashov, 2003; Lynch, 2010; Campbell and Eichler, 2013; Ramu et al., 2013; Besenbacher et al., 2015; Zhao et al., 2018). This mutation rate is one order of magnitude lower than that for SNP and five orders of magnitude lower than that for STR. Therefore, Indels combine the advantages of SNP and STR. Multi-Indels increase their polymorphism while retaining the advantages of Indels. We used Haploview to initially screen haplotype frequency. Since Haploview can only recognize biallelic alleles and biallelic loci are the most prevalent in Indels, this study investigated only biallelic Indels. We extracted 2,052,970 biallelic Indels from 22 autosomes in the 1000 Genomes Project using VCFtools. We further restricted the alleles according to their length. In theory, different amplicon lengths represent different haplotypes, so haplotype polymorphism can be determined according to allele frequency. In addition, the allele frequencies of SNP/InDel vary significantly among different populations. When applied to individual identification in forensic cases, population-specific allele frequencies are necessary (Oldoni et al., 2018). In our study, the application in the Chinese population is temporarily considered, so only the CHB and CHS population in the 1000 Genomes Project phase 3 are used as the source of screening candidate markers.

As a result, the frequency of some multi-Indel markers differed from the theoretical data obtained by the 1000 genome project database using Haploview (Supplementary Tables S1, S3). According to the law of free combination, three single markers with linkage equilibrium should display eight different haplotypes. A haplotype with a minimum frequency of 0.001 can be obtained using Haploview calculations. However, we found three multi-Indel markers (mh04zl001, mh10zl002, and mh18zl001) with three Indels having only two different haplotypes as two alleles, which might be related to the complete LD between closely adjacent markers (distances were 17, 38, and 16 bp, respectively). Additionally, seven multi-Indel markers were inconsistent with the theoretical amplicon length, and the haplotype frequency was also different. We verified the homozygous samples of each marker by Sanger sequencing, especially each amplicon that was inconsistent with the theoretical length. Although our screen limited the existence of other Indels with MAF >0.005 in this range, mh02zl001 and mh02zl003 had 10 and 9 haplotypes, respectively, because additional Indels were detected in this range. In addition, according to the Sanger sequencing results, novel mutations were also found in the mh10zl001 and mh21zl001 loci, which caused the actual allele size and frequency to be inconsistent with the theoretical value. For the other two loci, mh03zl003 and mh21zl002, we did not find redundant mutations in homozygous samples that have been sequenced by Sanger, but there are also inconsistencies with alleles. These Indels were not included in the database because the goal of the 1000 Genomes Project is to capture the most common human genetic variations (Bergstrom et al., 2020). The development and progress of sequencing technology allows the collection of more varied information.

Our multi-Indel multiplex panel has many advantages. We designed one pair of primers for each multi-Indel marker and one PCR amplicon and one CE run for genotyping. The elimination of sequences with 2–15 nucleotide tandem repeats improved genotyping accuracy and avoided stutter, which is a benefit when analyzing mixtures. Low mutation rates are highly significant in paternity testing, but our results showed that our panel could only serve as an effective supplement to STR, because the PE was not high enough (Huang et al., 2014; Gao et al., 2015; Zhao et al., 2018).

A generalized microhaplotype is essentially a set of all variants in a short fragment, namely multi-variants, which have higher polymorphism. The MPS technology can directly obtain sequences within the read length range, and thus directly determine the phase of a haplotype. Currently, the CE platform is more prevalent in forensic laboratories, so multi-Indels have other potential applications. With the future popularization of MPS, the application of generalized microhaplotypes will become more widespread.

## Conclusion

In our research, we proposed that the generalized microhaplotype is essentially a collection of all variants in a very short fragment (200 bp), that is, multi-variants with high polymorphism. At present, as the CE platform was widely used in all forensic genetic laboratories, a method based on the CE platform is described in this study. This method can simultaneously detect 18 microhaplotype markers consisting of three or more Indels with different insertion or deletion lengths in the range of less than 200 bp. Our multi-InDel microhaplotypes panel have shorter fragments than conventional STR markers, and have more potential in forensics considering the degraded DNA. In addition, multi-InDel microhaplotypes do not generate stutter involved with PCR amplification, which have more potential in forensics considering the mixture of DNA from two or more individuals. Finally, multi-InDel microhaplotypes offer a much lower mutation rate than STR markers, and it can be used as supplementary in paternity cases with STR mutation. And the results of combined power of discrimination (CPD) (0.999999999997234) certified the usefulness of our panel for forensic personal identification. But our results also showed that our panel can only be used as an effective supplement to STR, because the CPE (0.9984) is not high enough. Therefore, microhaplotypes consisting of three or more Indels which can be resolved by CE platform have great application potential in forensic genetics.

## Data Availability Statement

The datasets generated for this study can be found in the Figshare: https://doi.org/10.6084/m9.figshare.12924551.v2.

## Ethics Statement

The studies involving human participants were reviewed and approved by Ethical Committee of the Sichuan University. The patients/participants provided their written informed consent to participate in this study.

## Author Contributions

SQ, WL, and LZ designed this study. SQ, ML, and JX wrote the manuscript. YL conducted sample collection. SQ, JZ, and RZ conducted the experiment. SQ, LW, HJ, and LaZ analyzed the results. All authors reviewed the manuscript. All authors contributed to the article and approved the submitted version.

## Conflict of Interest

The authors declare that the research was conducted in the absence of any commercial or financial relationships that could be construed as a potential conflict of interest.

## Footnotes

1 https://vcftools.github.io/man_latest.html

2 http://primer3.ut.ee/

3 http://www.cstl.nist.gov/biotech/strbase/AutoDimerHomepage/AutoDimerProgramHomepage.htm

4 https://www.ncbi.nlm.nih.gov/tools/primer-blast/index.cgi

## Supplementary Material

Supplementary Figure 1 Representative electropherogram of control DNA M308 amplified at 1 ng.
