# Supplementary figures and images for: Multi-Indel: A Microhaplotype Marker Can Be Typed Using Capillary Electrophoresis Platforms
Source: Front Genet. 2020 Oct 23;11:567082. doi: 10.3389/fgene.2020.567082 (PMC7649793; doi:10.3389/fgene.2020.567082)

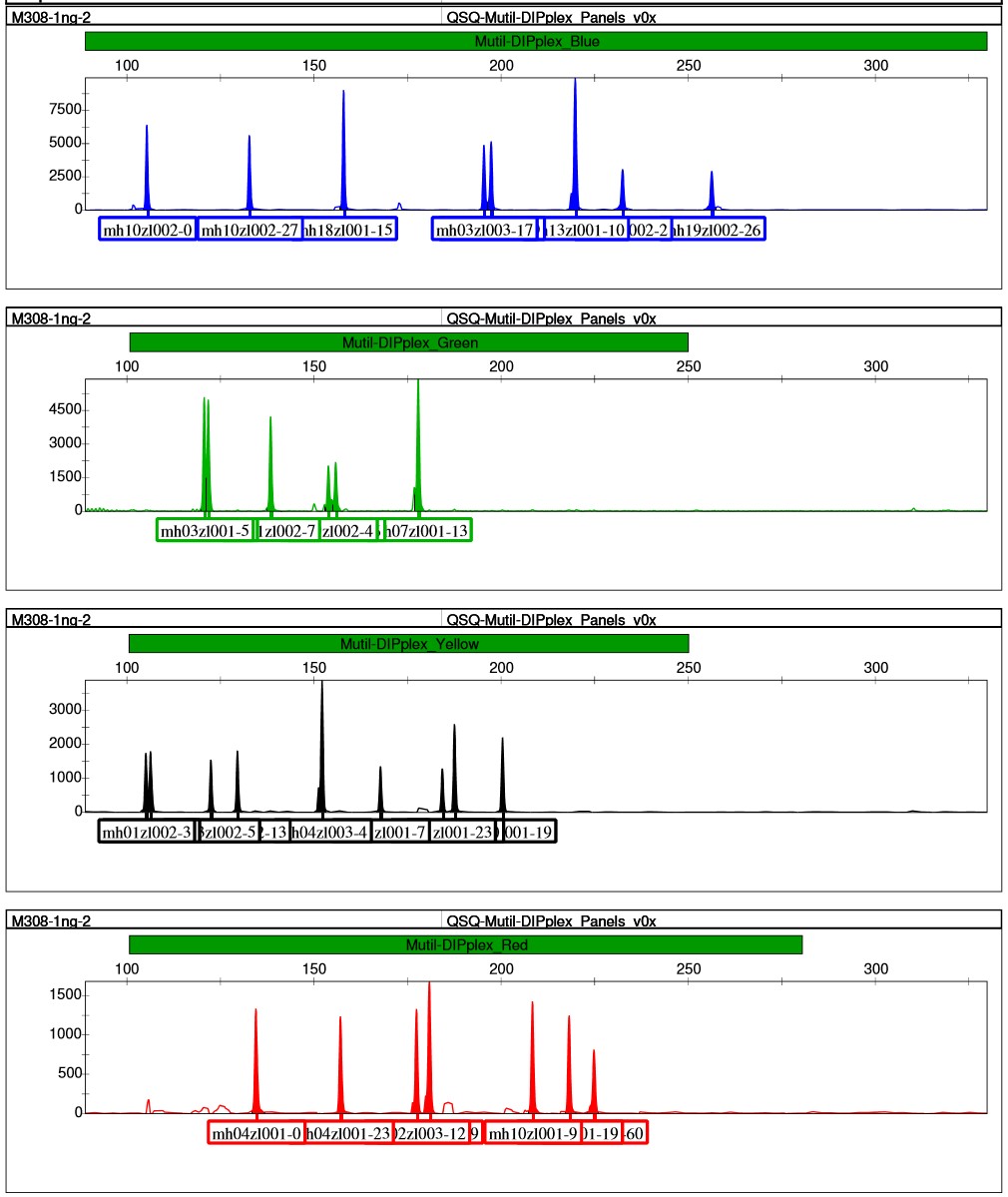

Supplement: Supplementary Figure 1 — Representative electropherogram of control DNA M308 amplified at 1 ng. [file Image_1.JPEG]
