# Supplementary material for: Multi-Indel: A Microhaplotype Marker Can Be Typed Using Capillary Electrophoresis Platforms
Source: Front Genet. 2020 Oct 23;11:567082. doi: 10.3389/fgene.2020.567082 (PMC7649793; doi:10.3389/fgene.2020.567082)

**Supplementary Data**

**The general information, capillary electrophoresis (CE) and Sanger sequencing diagrams of 18 singleplex PCR reactions and detection.**

| **ID** | **Microhaplotype** | **GRCh37** | **rs-Number dbSNP** | **Extent in bp** | **Allele1/Allele2** | **Insertion allele length** | **Primer sequences (label)** | **The actual amplicon size (bp)** |
| --- | --- | --- | --- | --- | --- | --- | --- | --- |
| 1 | mh01zl001 | 2029533 | rs368828322 | 16 | A/AG | 1 | GGCGGGGTGAATAGTTTGAC (ROX) | 179 |
|  |  | 2029539 | rs372567620 |  | A/AAGGTCAGAGC | 10 | TCAGTAAACAACCCCTGCCT |  |
|  |  | 2029549 | rs148361309 |  | C/CAGGTGACCAGGAGTGACTA | 19 |  |  |


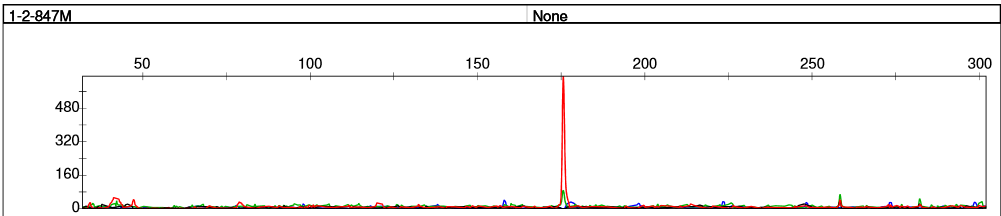


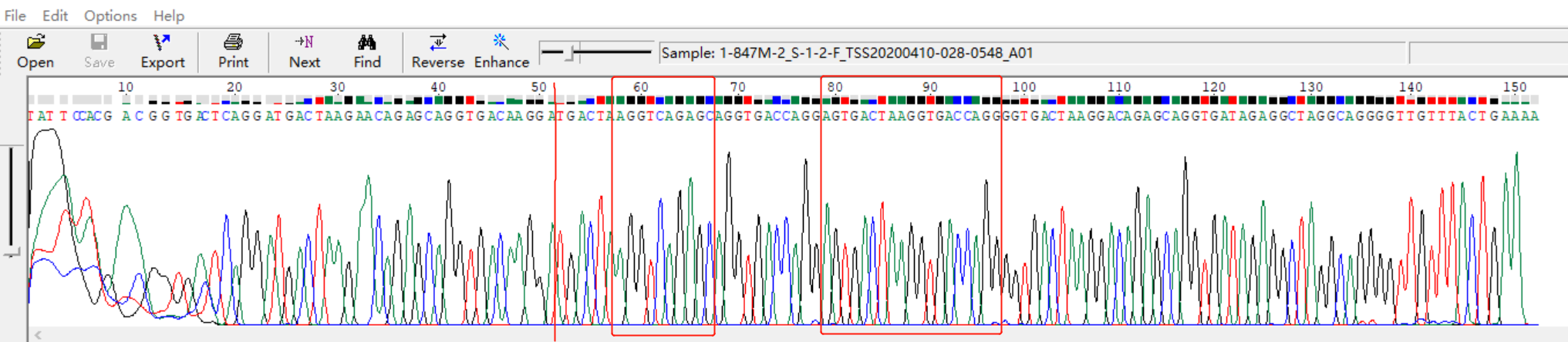


| **ID** | **Microhaplotype** | **GRCh37** | **rs-Number dbSNP** | **Extent in bp** | **Allele1/Allele2** | **Insertion allele length** | **Primer sequences (label)** | **The actual amplicon size (bp)** |
| --- | --- | --- | --- | --- | --- | --- | --- | --- |
| 2 | mh01zl002 | 100194878 | rs55796544 | 25 | C/CCT | 2 | TGTGCTCCTCTTTCTCACTAGT (TAMRA) | 107 |
|  |  | 100194896 | rs67810269 |  | C/CTGTA | 4 | TTAAGATGGTCAGGGCATCAG |  |
|  |  | 100194903 | rs71075445 |  | C/CT | 1 |  |  |


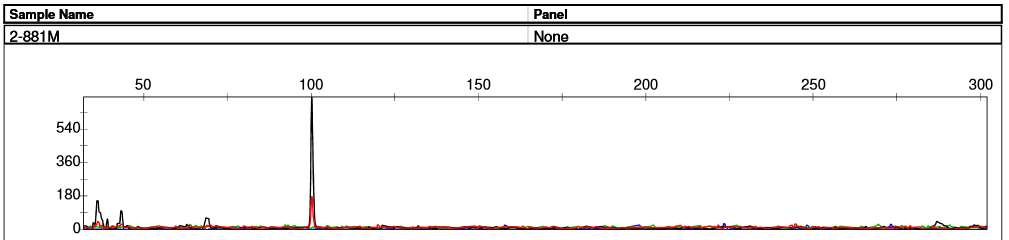


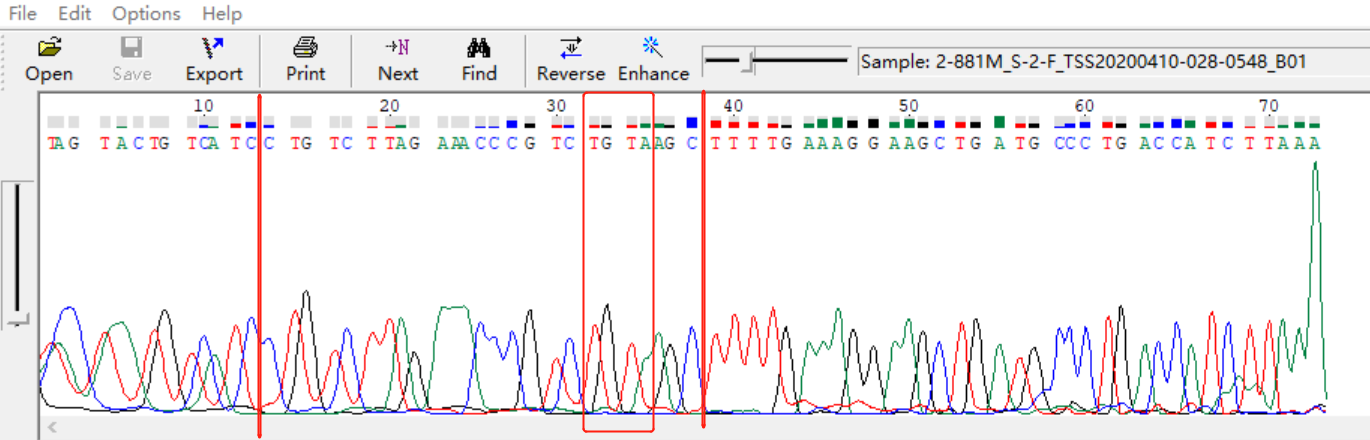

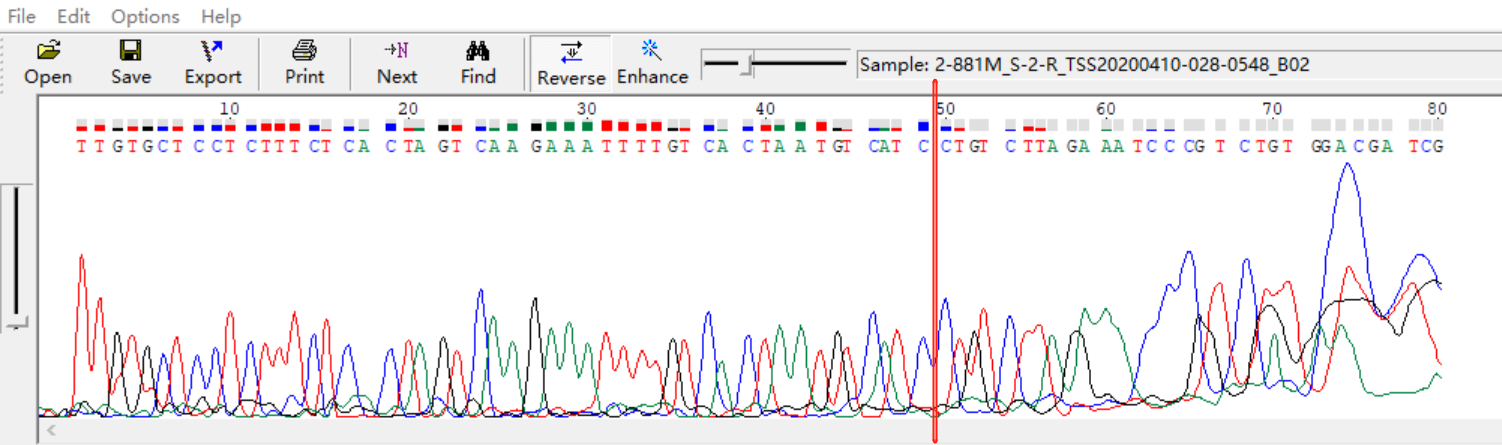


| **ID** | **Microhaplotype** | **GRCh37** | **rs-Number dbSNP** | **Extent in bp** | **Allele1/Allele2** | **Insertion allele length** | **Primer sequences (label)** | **The actual amplicon size (bp)** |
| --- | --- | --- | --- | --- | --- | --- | --- | --- |
| 5 | mh03zl001 | 73878996 | rs34404453 | 47 | T/TC | 1 | TGATTCTTCCTTACTCCTCCAAAG (HEX) | 126 |
|  |  | 73879030 | rs149171688 |  | A/AAATAT | 5 | GGCAACAGAATAAGACTCCGTT |  |
|  |  | 73879043 | rs34483288 |  | A/AATT | 3 |  |  |


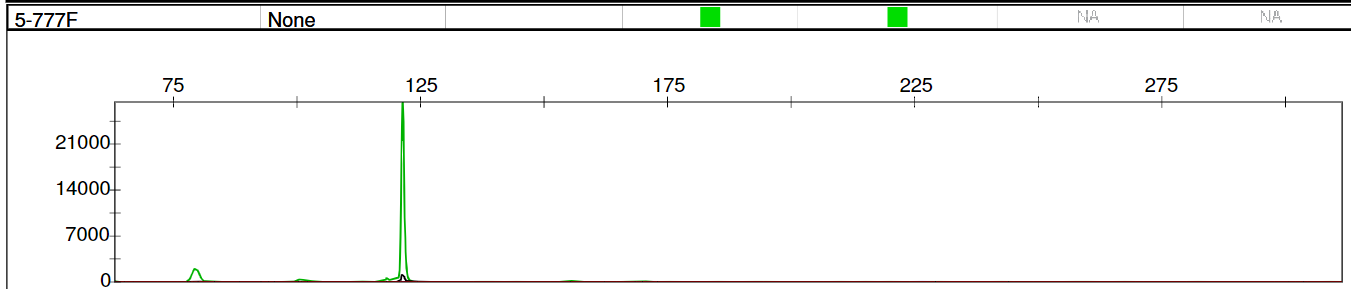


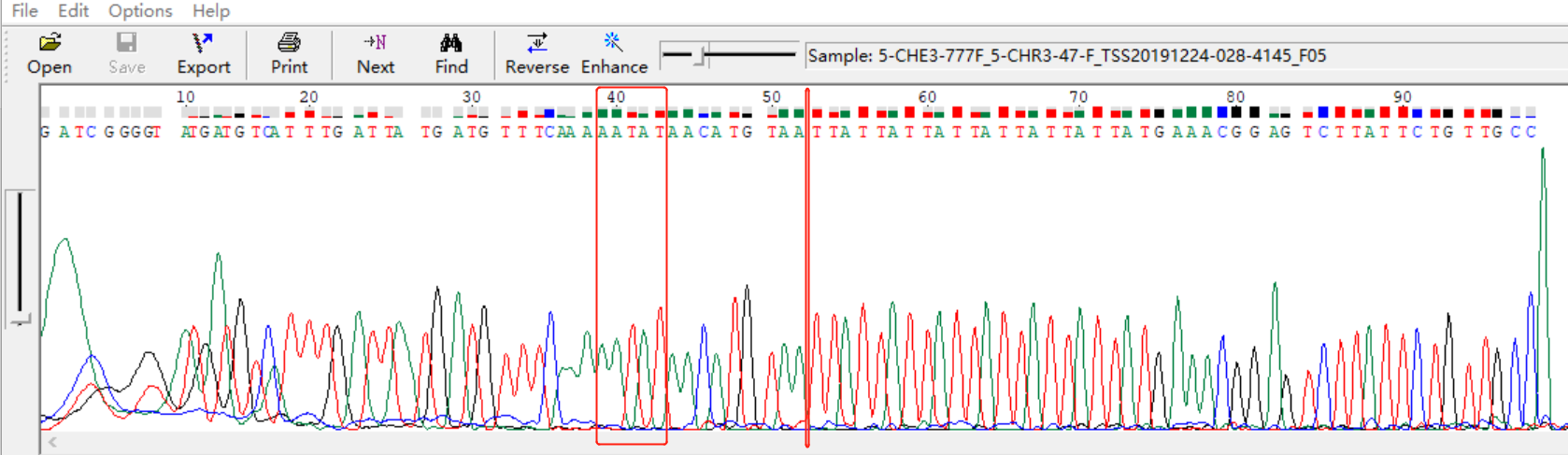

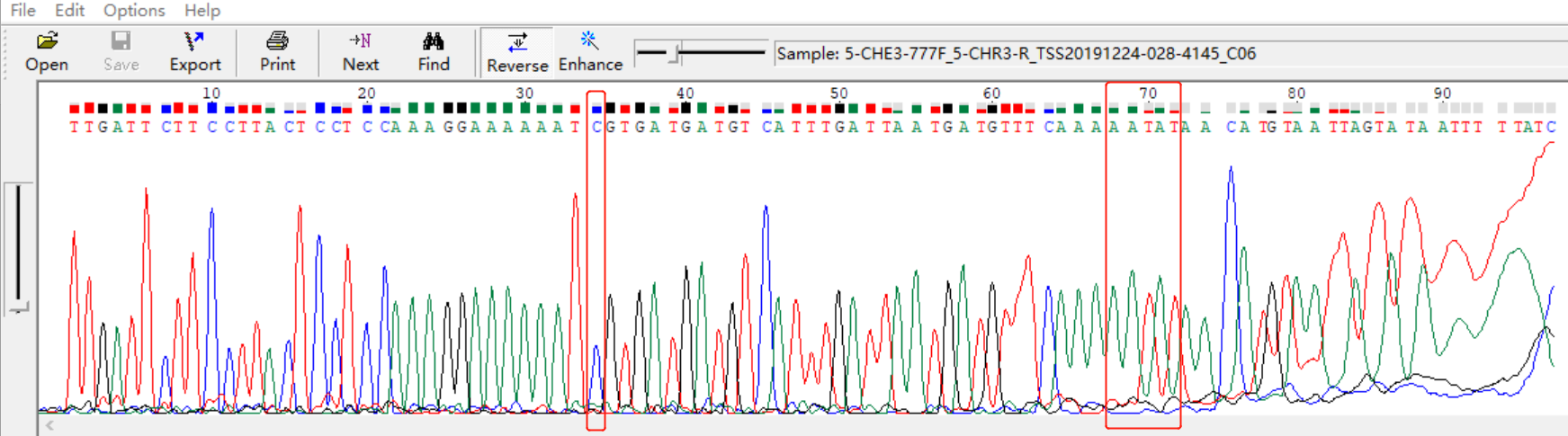


| **ID** | **Microhaplotype** | **GRCh37** | **rs-Number dbSNP** | **Extent in bp** | **Allele1/Allele2** | **Insertion allele length** | **Primer sequences (label)** | **The actual amplicon size (bp)** |
| --- | --- | --- | --- | --- | --- | --- | --- | --- |
| 6 | mh03zl002 | 87352688 | rs200679094 | 48 | G/GAAATCTAAATAT | 12 | ACCATCTACATTTTCCCTGTAAA | 130 |
|  |  | 87352693 | rs370444413 |  | A/AGGTG | 4 | GCTGGGTCATCGCCATTTT (TAMRA) |  |
|  |  | 87352736 | rs74604190 |  | T/TA | 1 |  |  |


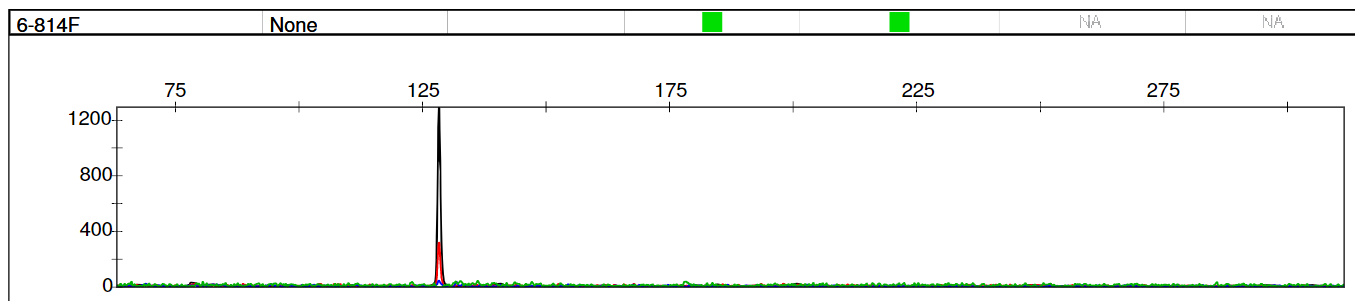


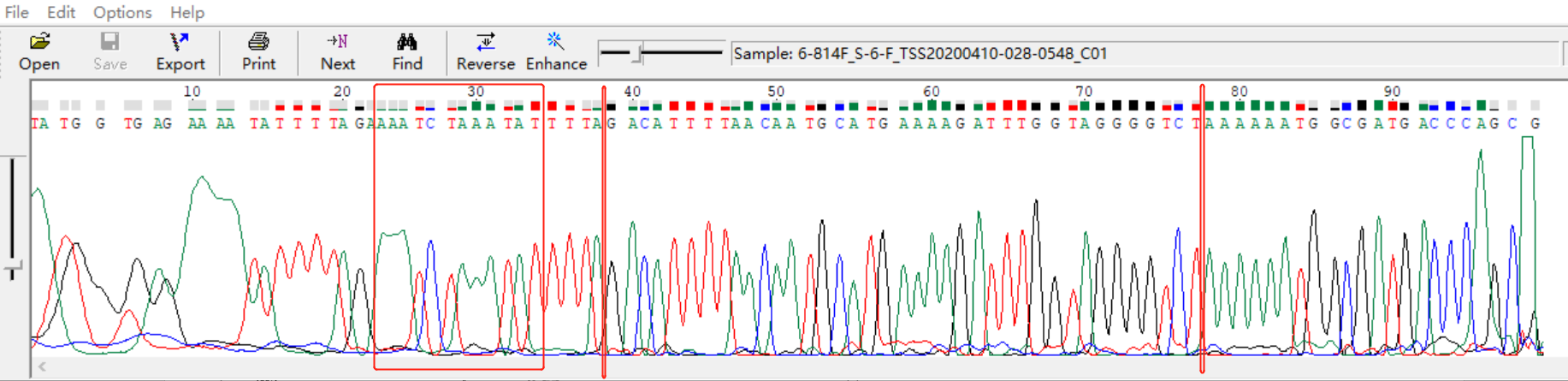


| **ID** | **Microhaplotype** | **GRCh37** | **rs-Number dbSNP** | **Extent in bp** | **Allele1/Allele2** | **Insertion allele length** | **Primer sequences (label)** | **The actual amplicon size (bp)** |
| --- | --- | --- | --- | --- | --- | --- | --- | --- |
| 7 | mh04zl001 | 18391312 | rs11282557 | 17 | G/GACAGTATTT | 9 | CCTTGTTGCTGCAGTAGAAAAT (ROX) | 135 |
|  |  | 18391316 | rs58595156 |  | G/GGAAAAATTGCT | 11 | TGATCACTTAAGTTCGATGAAAGAA |  |
|  |  | 18391329 | rs372089291 |  | A/ATTCTCCTAAATT | 12 |  |  |


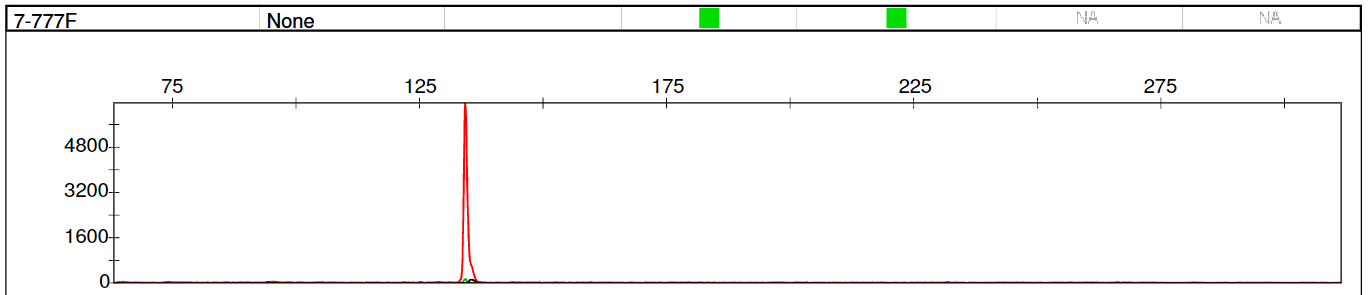


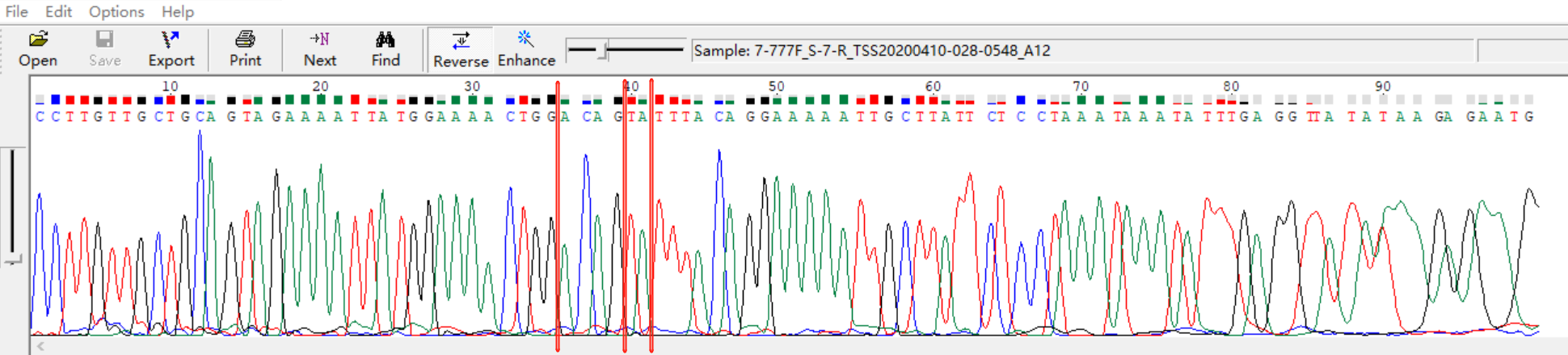


| **ID** | **Microhaplotype** | **GRCh37** | **rs-Number dbSNP** | **Extent in bp** | **Allele1/Allele2** | **Insertion allele length** | **Primer sequences (label)** | **The actual amplicon size (bp)** |
| --- | --- | --- | --- | --- | --- | --- | --- | --- |
| 14 | mh10zl002 | 113582580 | rs143378119 | 38 | G/GAGAATACATTA | 11 | GAACAGAGTGTCATCCATTTTCT | 137 |
|  |  | 113582616 | rs370632025 |  | T/TTATGG | 5 | TCAGGCCAATCACACGTG (FAM) |  |
|  |  | 113582618 | rs571358799 |  | C/CAGGACTGGAAGGAGAATACAAT | 22 |  |  |


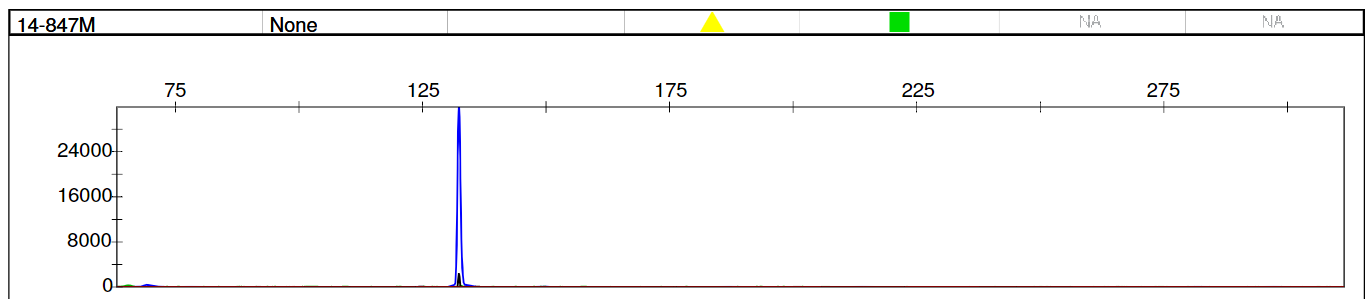


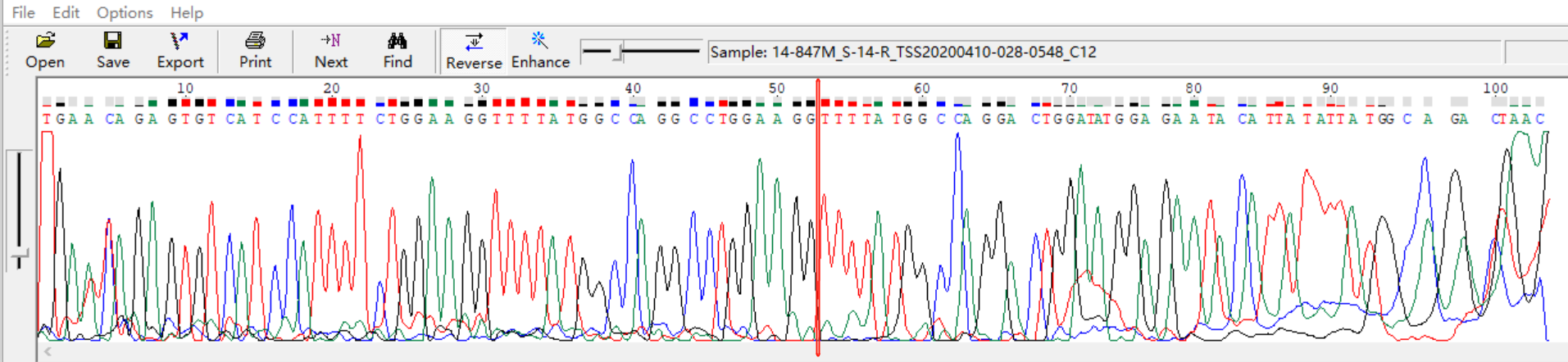

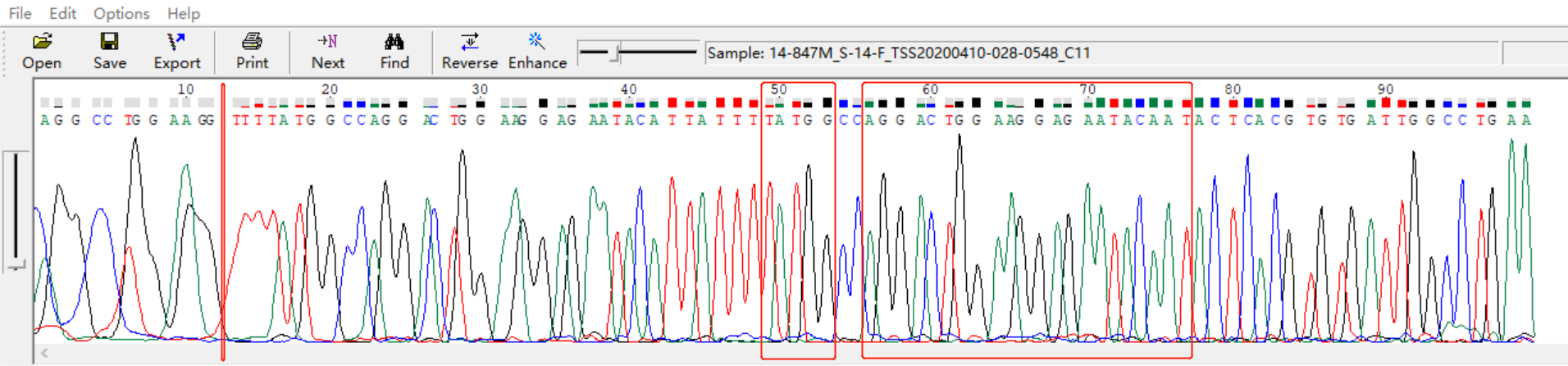


| **ID** | **Microhaplotype** | **GRCh37** | **rs-Number dbSNP** | **Extent in bp** | **Allele1/Allele2** | **Insertion allele length** | **Primer sequences (label)** | **The actual amplicon size (bp)** |
| --- | --- | --- | --- | --- | --- | --- | --- | --- |
| 17 | mh13zl001 | 25442007 | rs58303500 | 26 | G/GCAT | 3 | GCCGTGATCTTCCTGGGAA (FAM) | 220 |
|  |  | 25442012 | rs34156563 |  | A/AT | 1 | TAATGAGGGCTGGGGTGTTT |  |
|  |  | 25442033 | rs67523118 |  | A/ACTTATT | 6 |  |  |


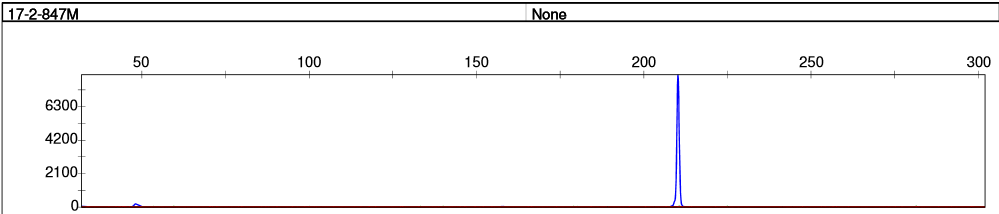


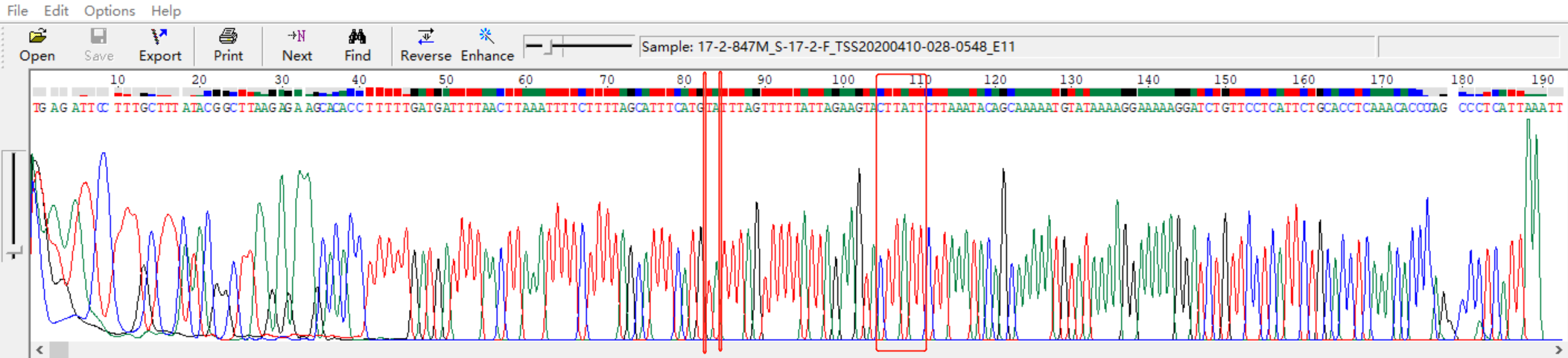


| **ID** | **Microhaplotype** | **GRCh37** | **rs-Number dbSNP** | **Extent in bp** | **Allele1/Allele2** | **Insertion allele length** | **Primer sequences (label)** | **The actual amplicon size (bp)** |
| --- | --- | --- | --- | --- | --- | --- | --- | --- |
| 21 | mh18zl001 | 61672654 | rs377195018 | 16 | A/AGAGGTGGGACC | 11 | GAATGCCGTCTTCCACCAAA (FAM) | 153 |
|  |  | 61672667 | rs59925455 |  | T/TTGGG | 4 | AGGGGCAAGGTAGTTCTCTG |  |
|  |  | 61672670 | rs201823781 |  | G/GAT | 2 |  |  |


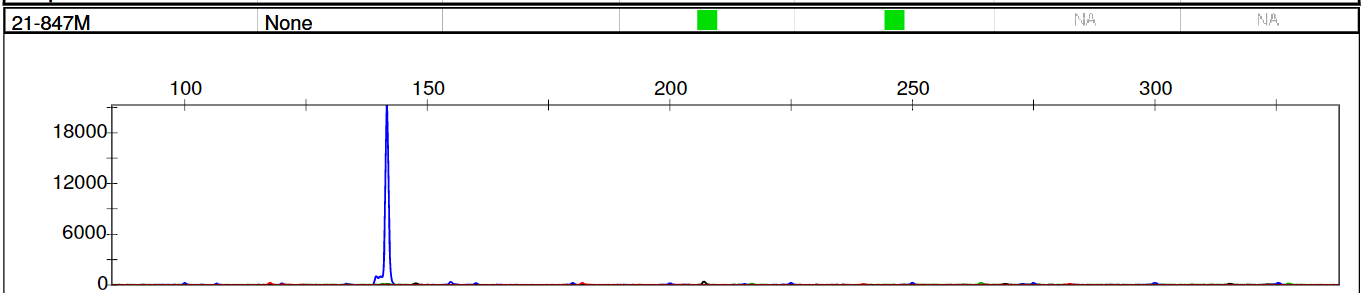


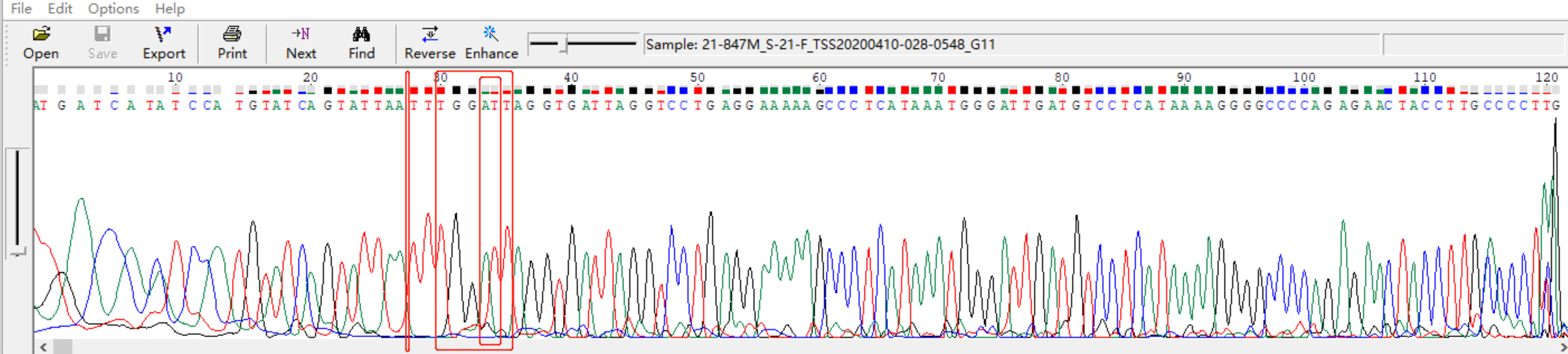


| **ID** | **Microhaplotype** | **GRCh37** | **rs-Number dbSNP** | **Extent in bp** | **Allele1/Allele2** | **Insertion allele length** | **Primer sequences (label)** | **The actual amplicon size (bp)** |
| --- | --- | --- | --- | --- | --- | --- | --- | --- |
| 22 | mh19zl001 | 490362 | rs138906215 | 87 | A/AAAAG | 4 | GGCGACAAGAGTGAAACTC | 162 |
|  |  | 490414 | rs35679623 |  | A/ACT | 2 | CTCAGCGTGAACAAAGAGTG (HEX) |  |
|  |  | 490449 | rs11283323 |  | C/CAATACTG | 7 |  |  |


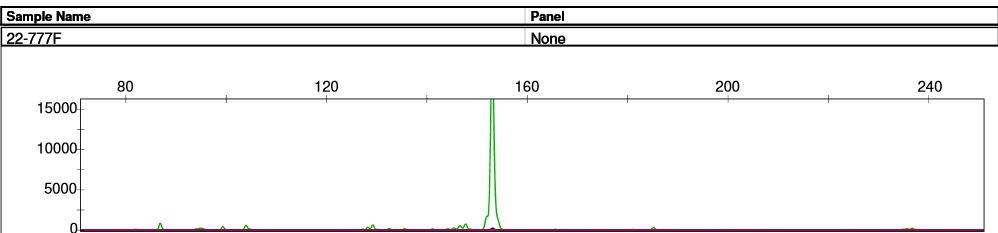


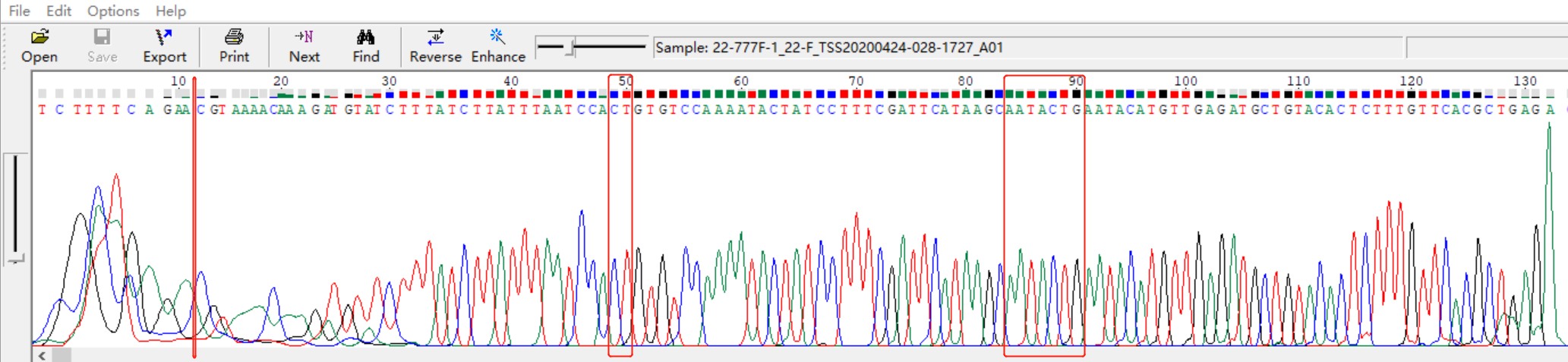

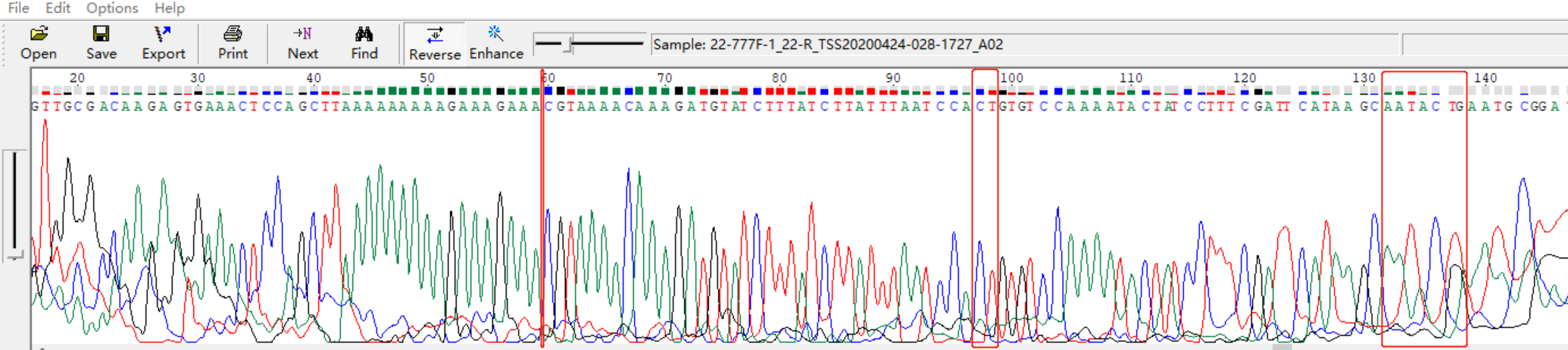


| **ID** | **Microhaplotype** | **GRCh37** | **rs-Number dbSNP** | **Extent in bp** | **Allele1/Allele2** | **Insertion allele length** | **Primer sequences (label)** | **The actual amplicon size (bp)** |
| --- | --- | --- | --- | --- | --- | --- | --- | --- |
| 23^*^ | mh19zl002 | 56039183 | rs543507020 | 85 | A/ATGCACACACTCACAATTGCACACACG | 26 | TGGACACACGCACACTGG (FAM) | 239 |
|  |  | 56039213 | rs559033587 |  | A/ACACT | 4 | TCTGTGCAAGTGTGAATGTCG |  |
|  |  | 56039268 | rs36127315 |  | G/GCA | 2 |  |  |

^*^The actual amplicon size at this locus is different from the theoretical amplicon size. When using the UCSC In-Silico PCR tool (http://genome.ucsc.edu/cgi-bin/hgPcr) to query the theoretical specificity of the primers, the results showed good specificity. When using Primer-BLAST tool (https://www.ncbi.nlm.nih.gov/tools/primer-blast/) to query primer specificity, it was found that two other non-specific products with similar fragment lengths appeared due to non-specific matching of the forward primer. But both non-specific products contain the target area. In the actual verification, the sequencing result showed that the actual amplified fragment was a non-specific product with an extra sequence. However, since it still contains the target sequence and does not affect the genotyping of the target sequence, the locus is still reserved.


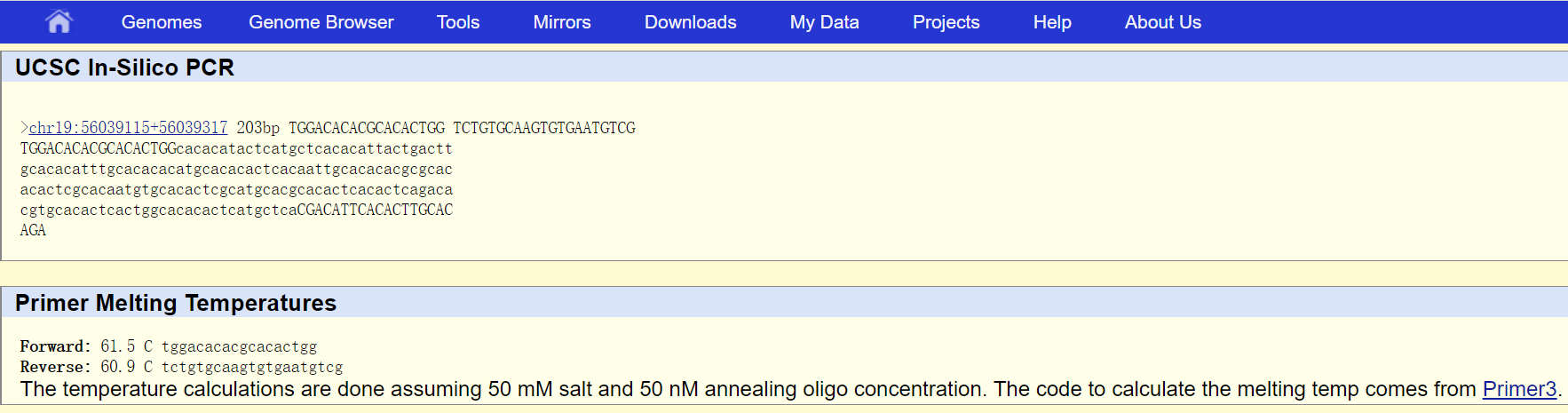


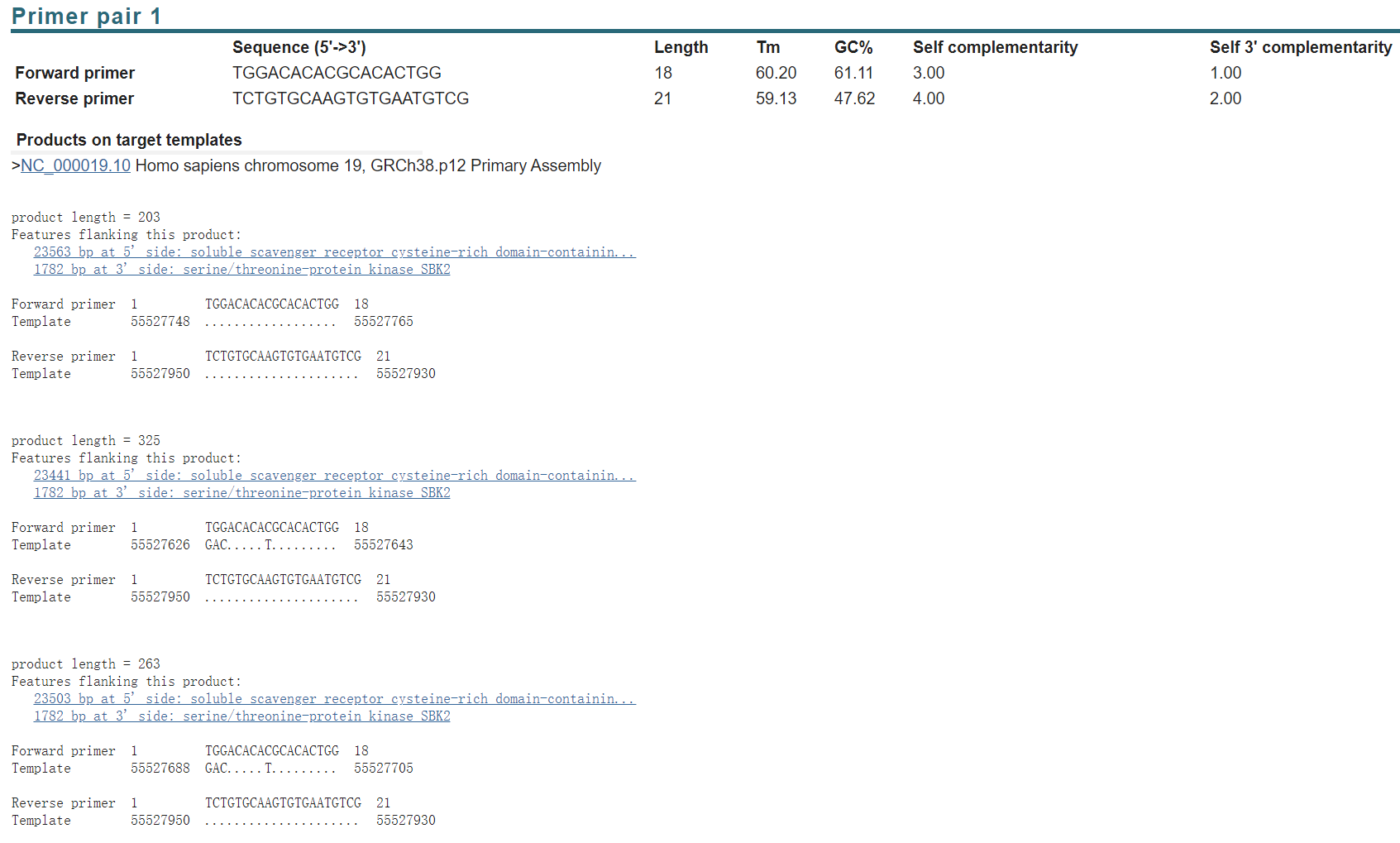


Sample 1


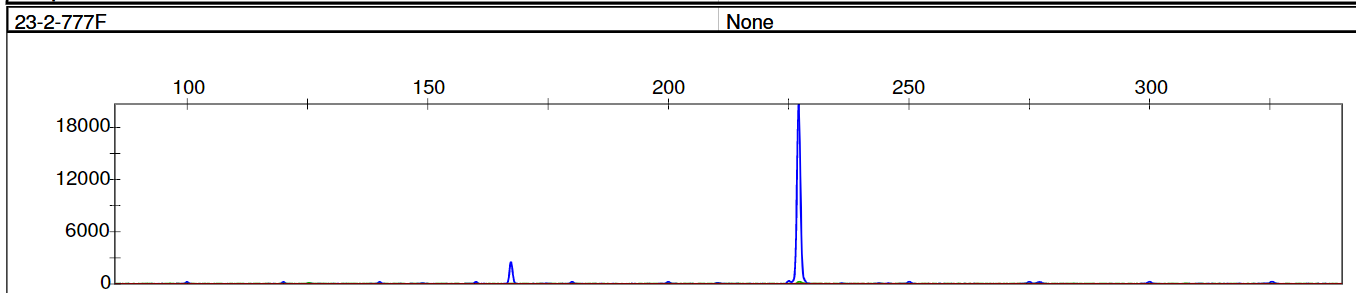


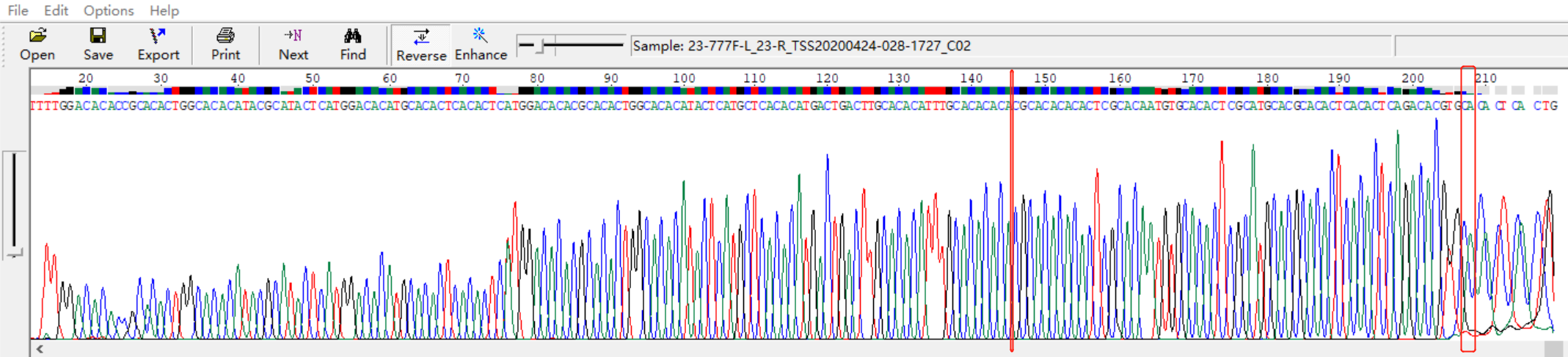


Sample 2


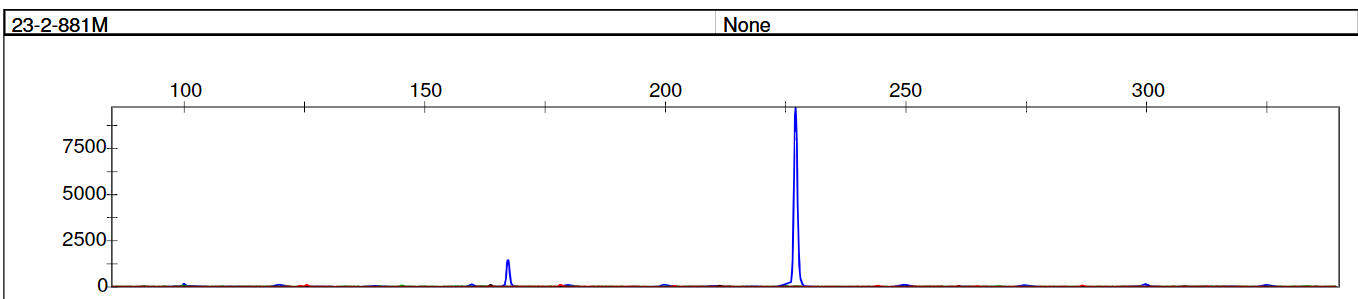


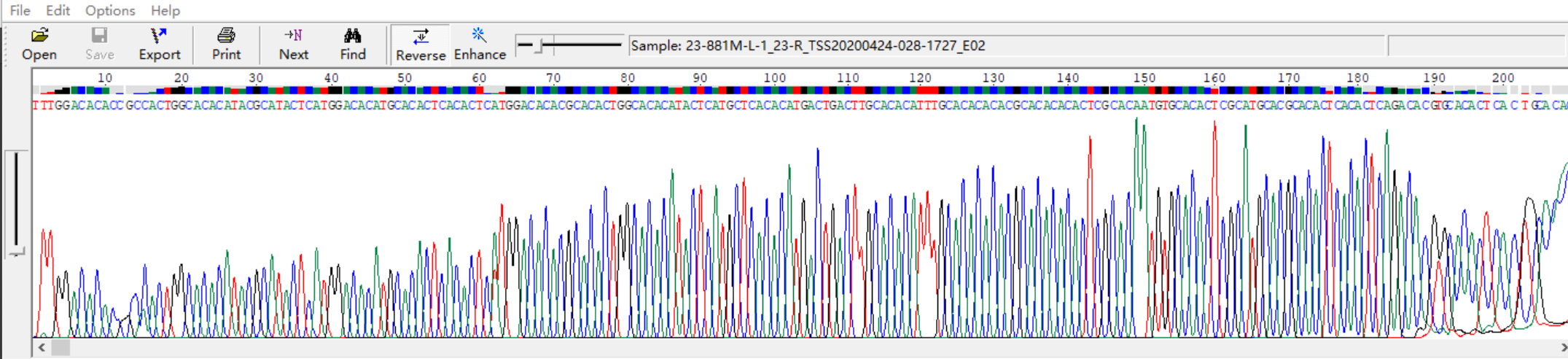


| **ID** | **Microhaplotype** | **GRCh37** | **rs-Number dbSNP** | **Extent in bp** | **Allele1/Allele2** | **Insertion allele length** | **Primer sequences (label)** | **The actual amplicon size (bp)** |
| --- | --- | --- | --- | --- | --- | --- | --- | --- |
| 24^*^ | mh21zl001 | 21261329 | rs562677243 | 5 | T/TTA | 2 | AGCAATGTGTTCACAGATACCA | 180 |
|  |  | 21261333 | rs576365249 |  | C/CG | 1 | AGGCCATGGAGAGGAGTAGA (TAMRA) |  |
|  |  | 21261334 | rs373980639 |  | G/GGGGACATTT | 9 |  |  |

^*^The actual amplicon size at this locus is different from the theoretical amplicon size. Because there is a variation (rs796855393) that is not included in the 1000 Genomes Project phase 3. However, this variation can be queried on the new dbSNP website. The variation type of this mutation is Indel (Insertion and Deletion), and the alleles are TTACCG/TCCAACTTTAGCCTGGGGACATTT. Therefore, the actual amplicon size is inconsistent with the theoretical amplicon size inferred from the 1000 Genomes Project phase 3.


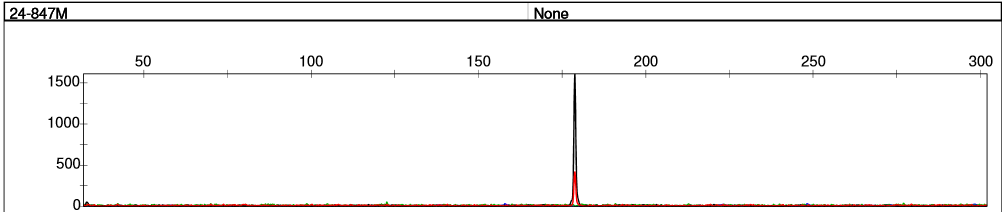


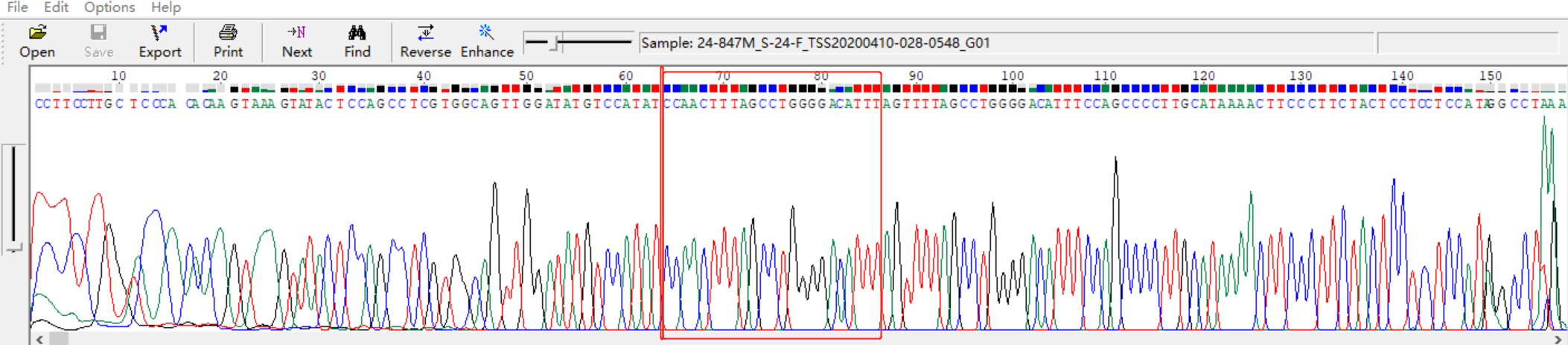


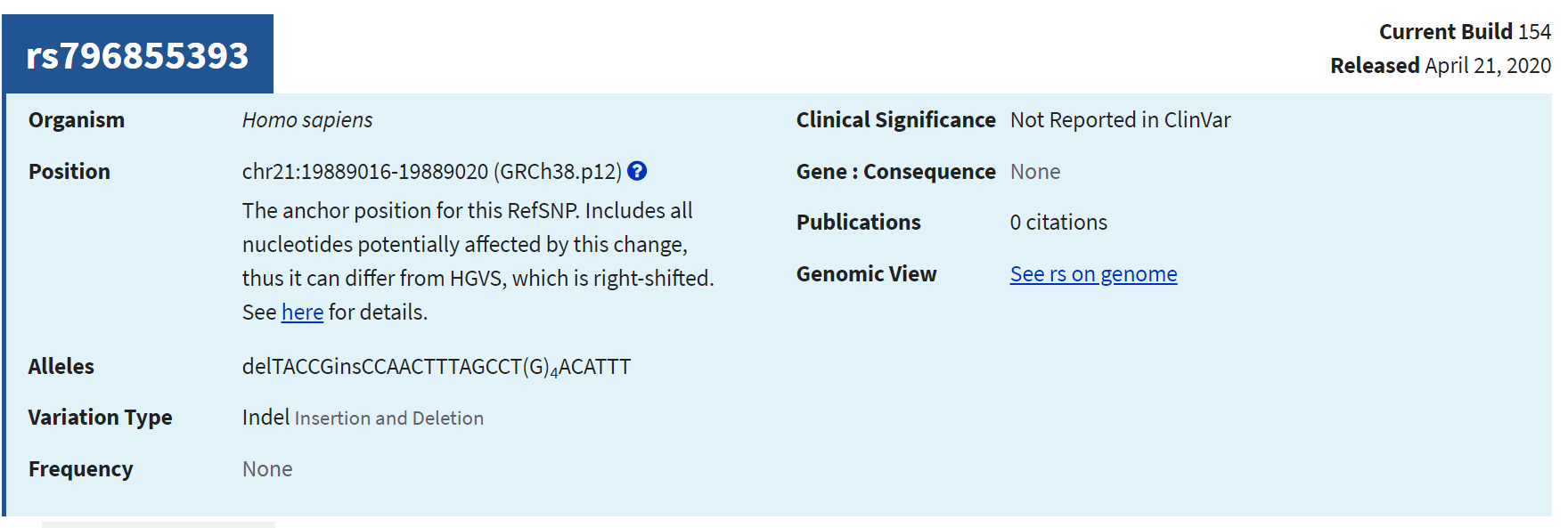


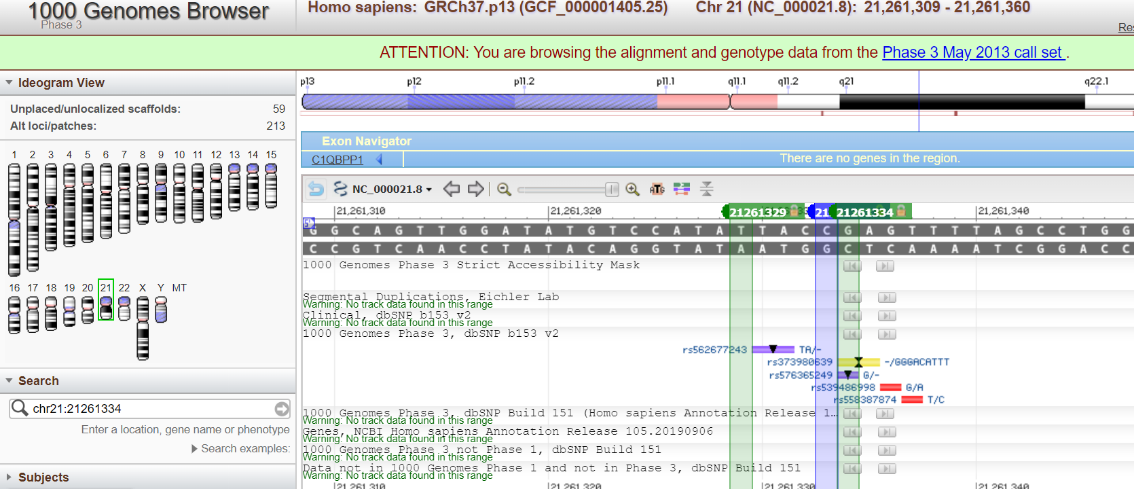

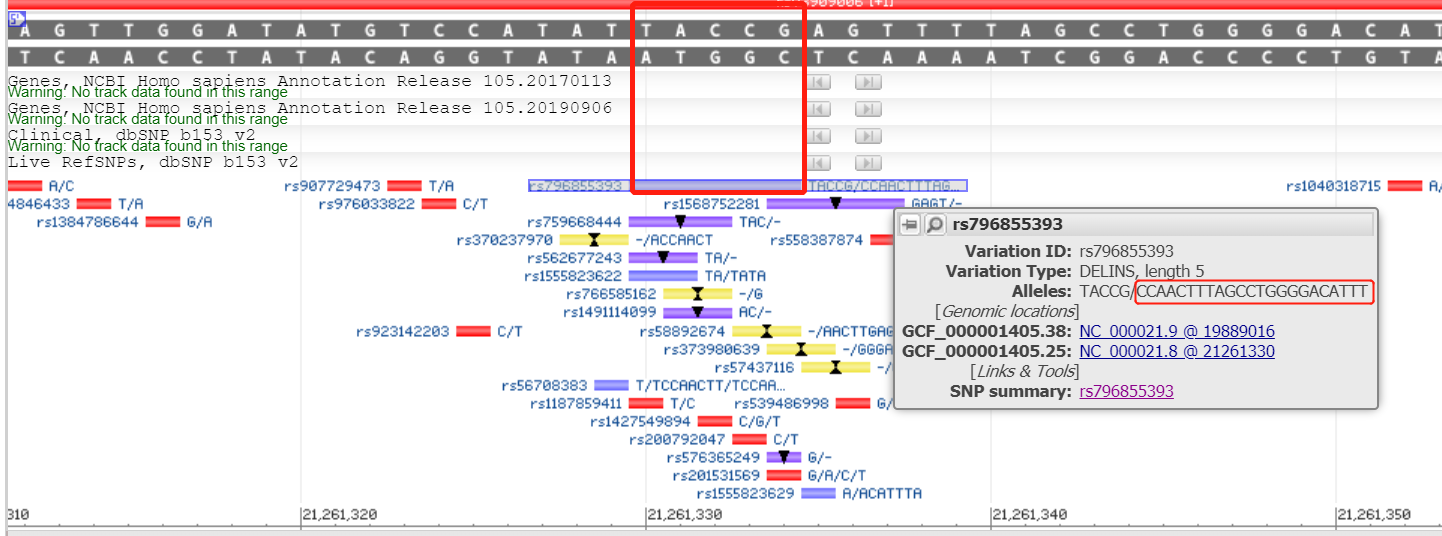


| **ID** | **Microhaplotype** | **GRCh37** | **rs-Number dbSNP** | **Extent in bp** | **Allele1/Allele2** | **Insertion allele length** | **Primer sequences (label)** | **The actual amplicon size (bp)** |
| --- | --- | --- | --- | --- | --- | --- | --- | --- |
| 29 | mh02zl001 | 30981778 | rs142363578 | 142 | C/CTTCT | 4 | CCCTTACTCCCTCTCGTCTTC (TAMRA) | 200 |
|  |  | 30981829 | rs144117237 |  | T/TTC | 2 | GGAGGGATGAAGGGAGGC |  |
|  |  | 30981920 | rs148016741 |  | C/CCCTCCCTCCCTCCCT | 15 |  |  |


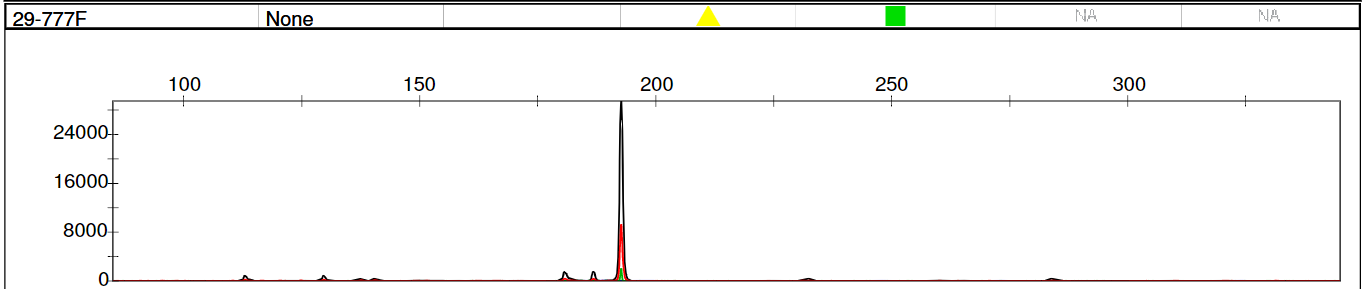


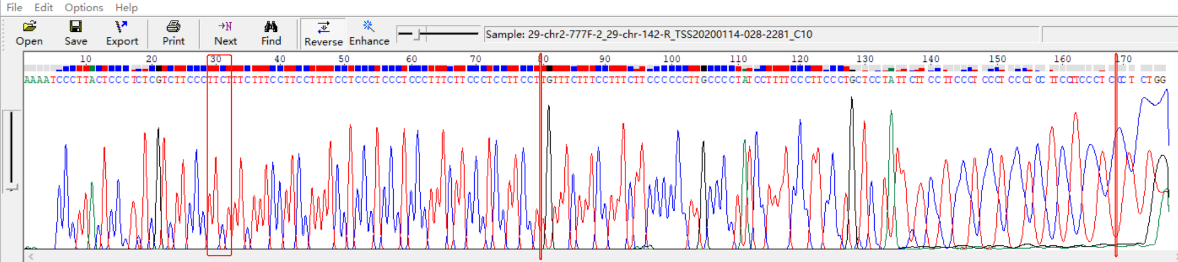

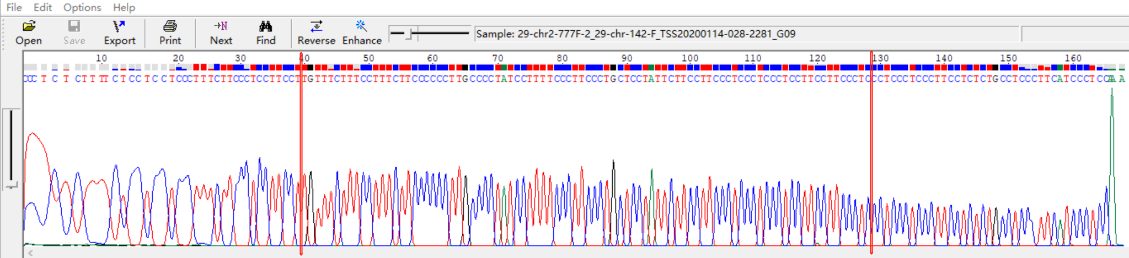


| **ID** | **Microhaplotype** | **GRCh37** | **rs-Number dbSNP** | **Extent in bp** | **Allele1/Allele2** | **Insertion allele length** | **Primer sequences (label)** | **The actual amplicon size (bp)** |
| --- | --- | --- | --- | --- | --- | --- | --- | --- |
| 30 | mh03zl003 | 163670527 | rs80013016 | 102 | T/TG | 1 | AGCTAGAGGTGTGTAGGCAA (FAM) | 199 |
|  |  | 163670601 | rs372207681 |  | C/CAGGTGCCAGCT | 11 | GCTTCTGCGTGACACTGC |  |
|  |  | 163670629 | rs111507567 |  | G/GCTGCTGCTTTGGGCAA | 16 |  |  |

GCC
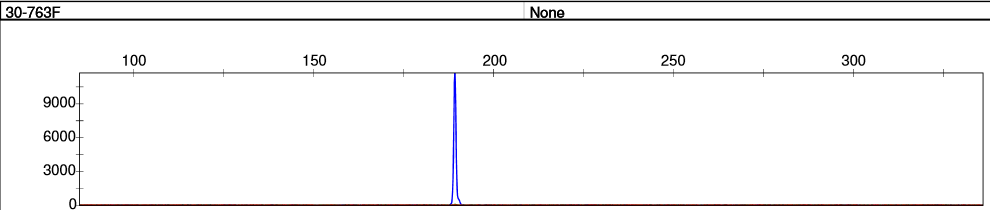


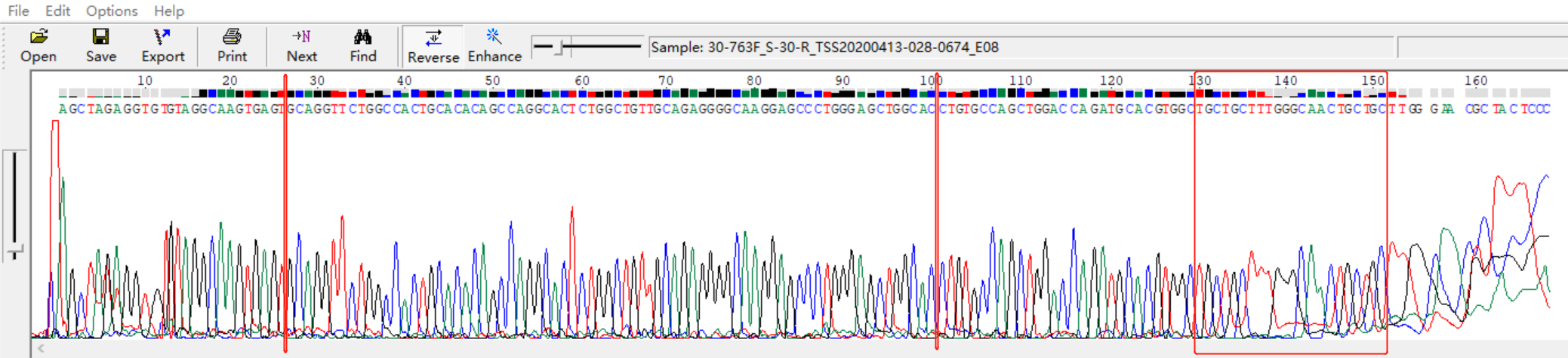


| **ID** | **Microhaplotype** | **GRCh37** | **rs-Number dbSNP** | **Extent in bp** | **Allele1/Allele2** | **Insertion allele length** | **Primer sequences (label)** | **The actual amplicon size (bp)** |
| --- | --- | --- | --- | --- | --- | --- | --- | --- |
| 33 | mh04zl003 | 187124231 | rs66502037 | 82 | C/CAT | 2 | TGCGCATATACACATACATAGATG | 157 |
|  |  | 187124238 | rs77222977 |  | G/GCACA | 4 | ATGTGTATGGGGTTGTGCAC (TAMRA) |  |
|  |  | 187124313 | rs71871946 |  | T/TTCATA | 5 |  |  |


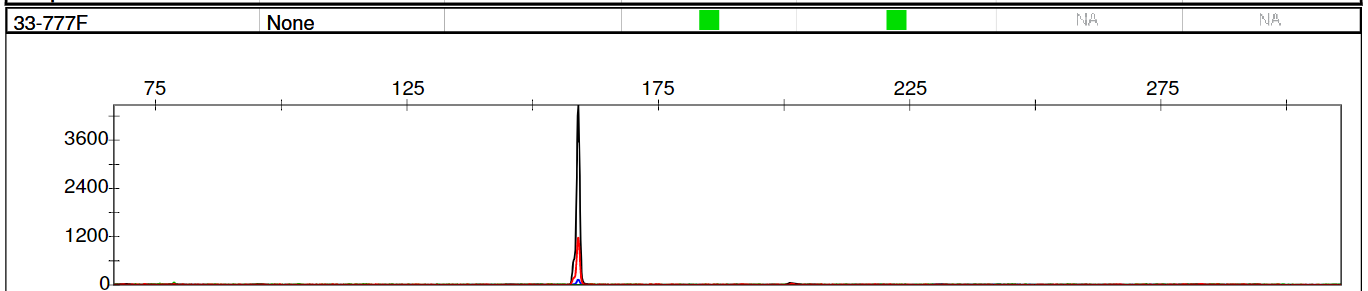


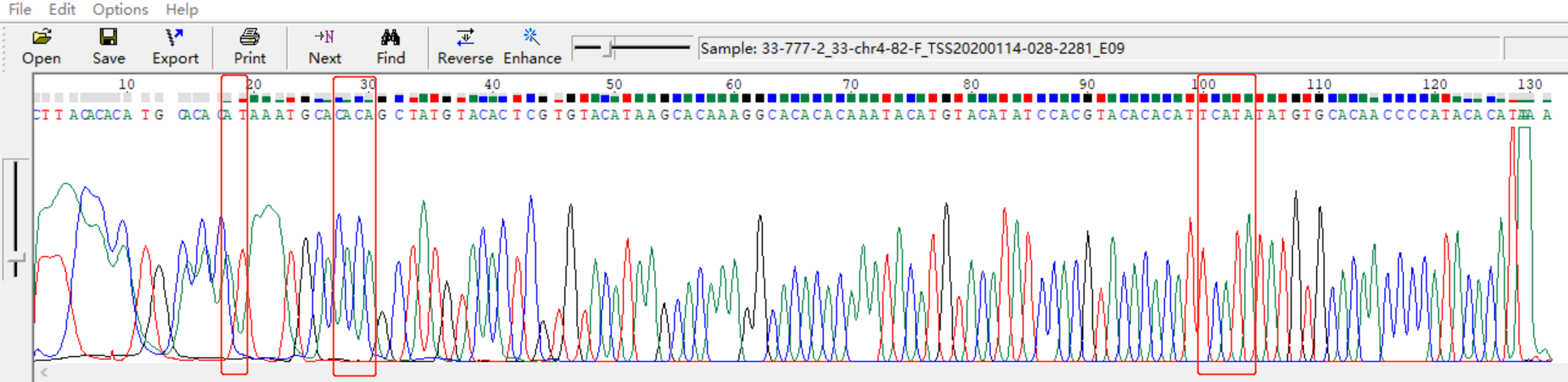


| **ID** | **Microhaplotype** | **GRCh37** | **rs-Number dbSNP** | **Extent in bp** | **Allele1/Allele2** | **Insertion allele length** | **Primer sequences (label)** | **The actual amplicon size (bp)** |
| --- | --- | --- | --- | --- | --- | --- | --- | --- |
| 35 | mh07zl001 | 57322877 | rs71053237 | 103 | C/CTAAATGAT | 8 | TTGTGGGGTGGCGGAAG | 179 |
|  |  | 57322974 | rs72447238 |  | T/TATA | 3 | GCACTGGATGGCACTCTTTT (HEX) |  |
|  |  | 57322980 | rs71053238 |  | A/AAT | 2 |  |  |


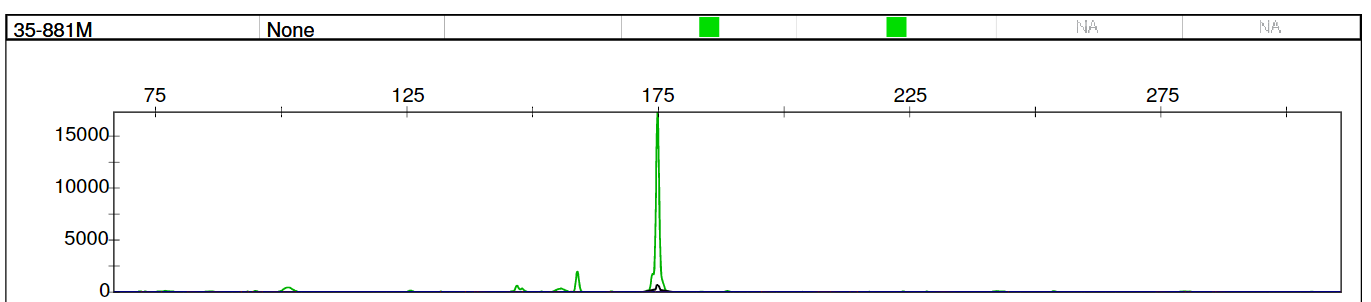


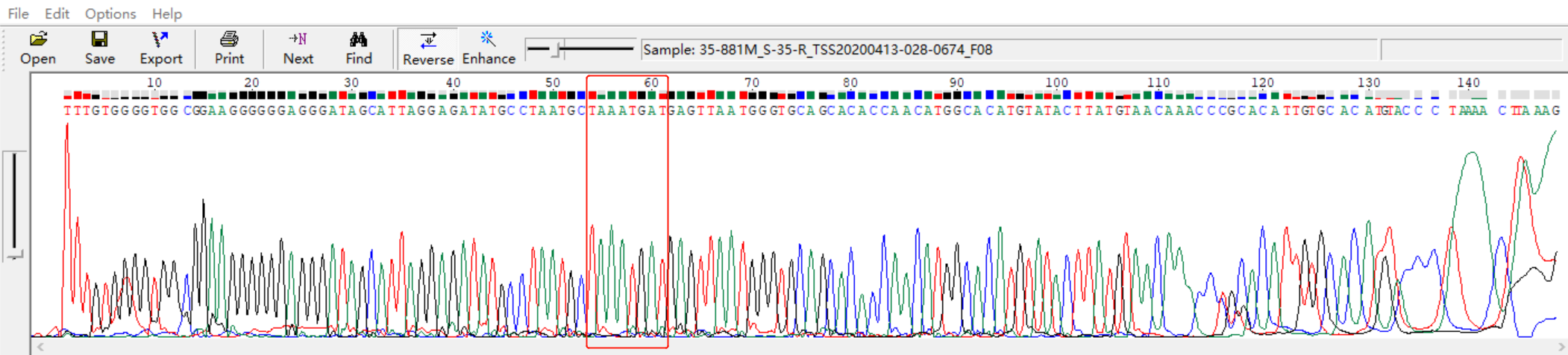

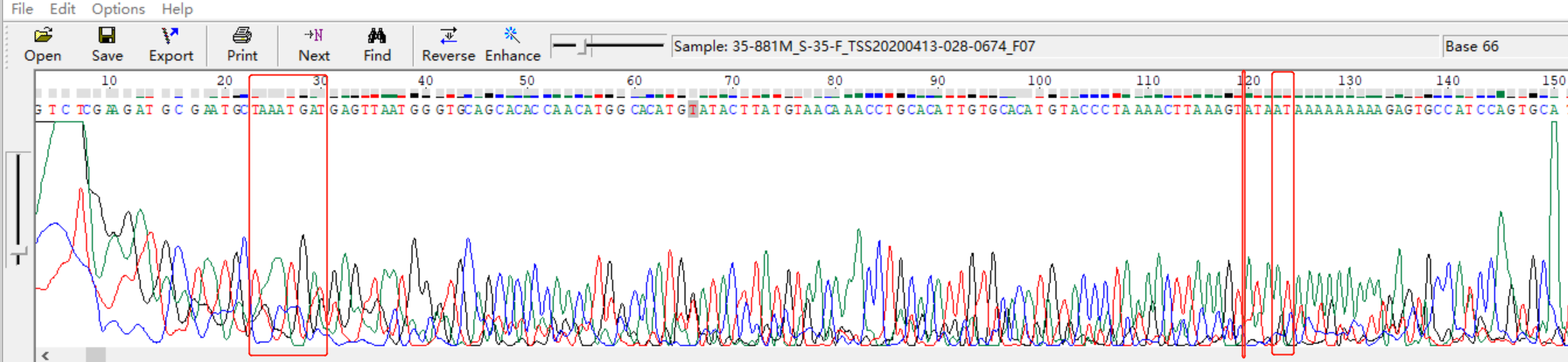


| **ID** | **Microhaplotype** | **GRCh37** | **rs-Number dbSNP** | **Extent in bp** | **Allele1/Allele2** | **Insertion allele length** | **Primer sequences (label)** | **The actual amplicon size (bp)** |
| --- | --- | --- | --- | --- | --- | --- | --- | --- |
| 38^*^ | mh10zl001 | 7140235 | rs145059123 | 72 | C/CA | 1 | ACACATTCACACATTCATTTAGACA (ROX) | 217 |
|  |  | 7140259 | rs539040996 |  | T/TAGACAC | 6 | TGGTGTGTGTGTATGCTAGTG |  |
|  |  | 7140307 | rs34860860 |  | T/TCA | 2 |  |  |

^*^The actual amplicon size at this locus is different from the theoretical amplicon size. Because there are two variations (rs751638126 and rs36127476) that are not included in the 1000 Genomes Project phase 3. However, these variations can be queried on the new dbSNP website. The variation type of rs751638126 is Indel, and the alleles are C/CAC. And the variation type of rs36127476 is Indel, and the alleles are A/AGACA. Therefore, the actual amplicon size is inconsistent with the theoretical amplicon size inferred from the 1000 Genomes Project phase 3.


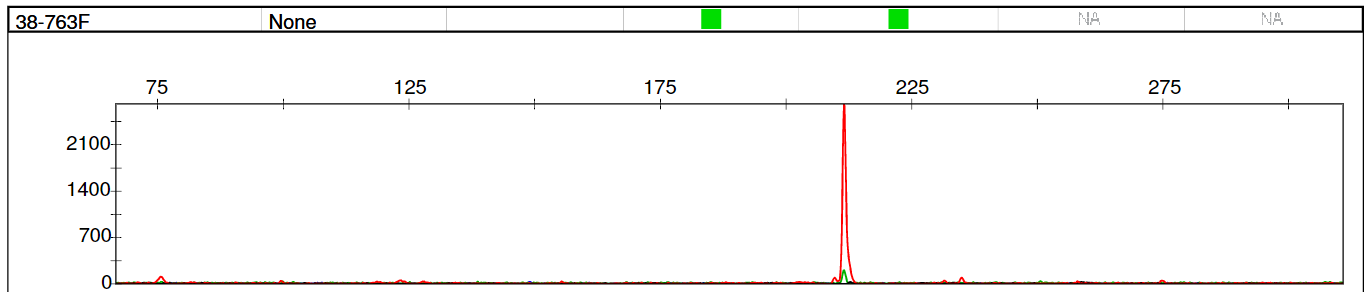


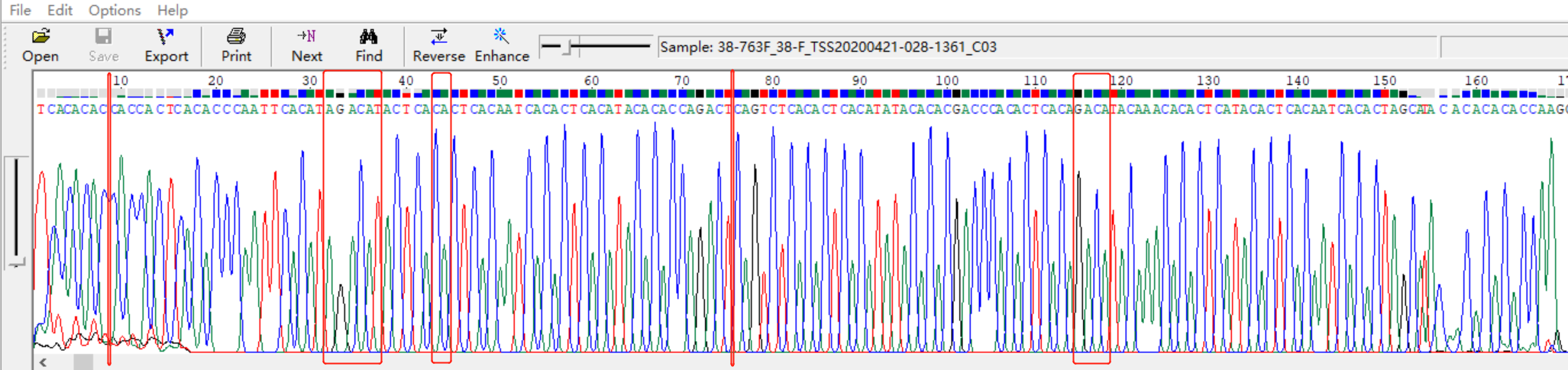


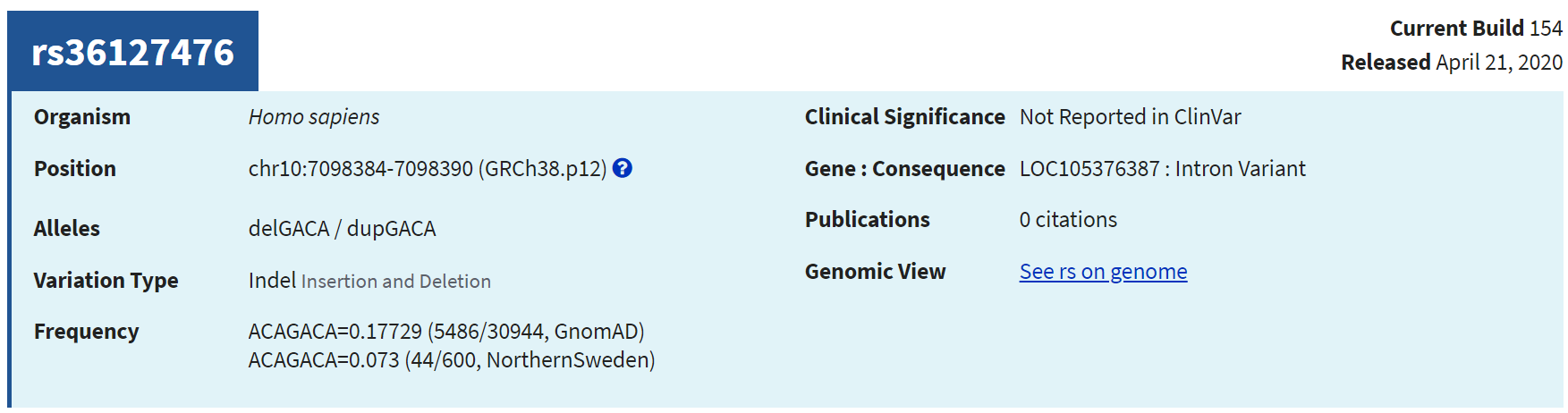


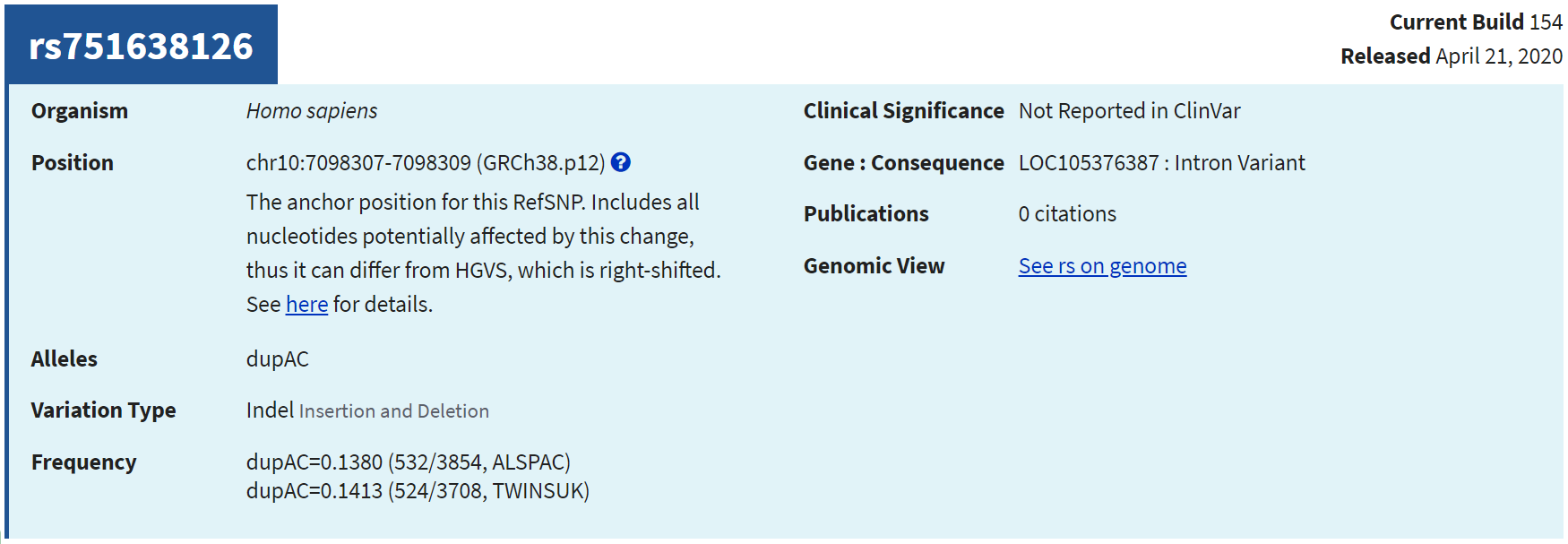


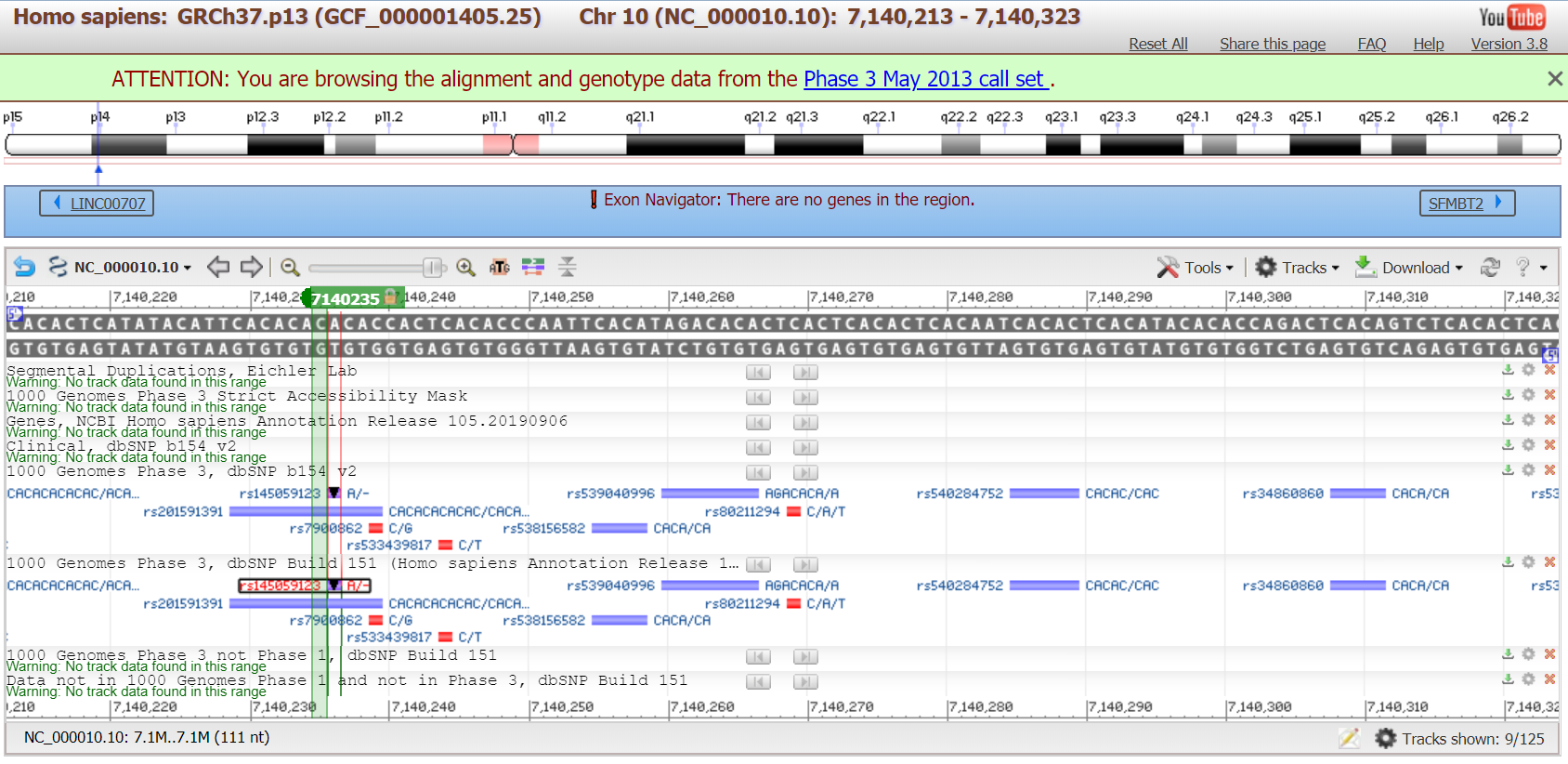


**
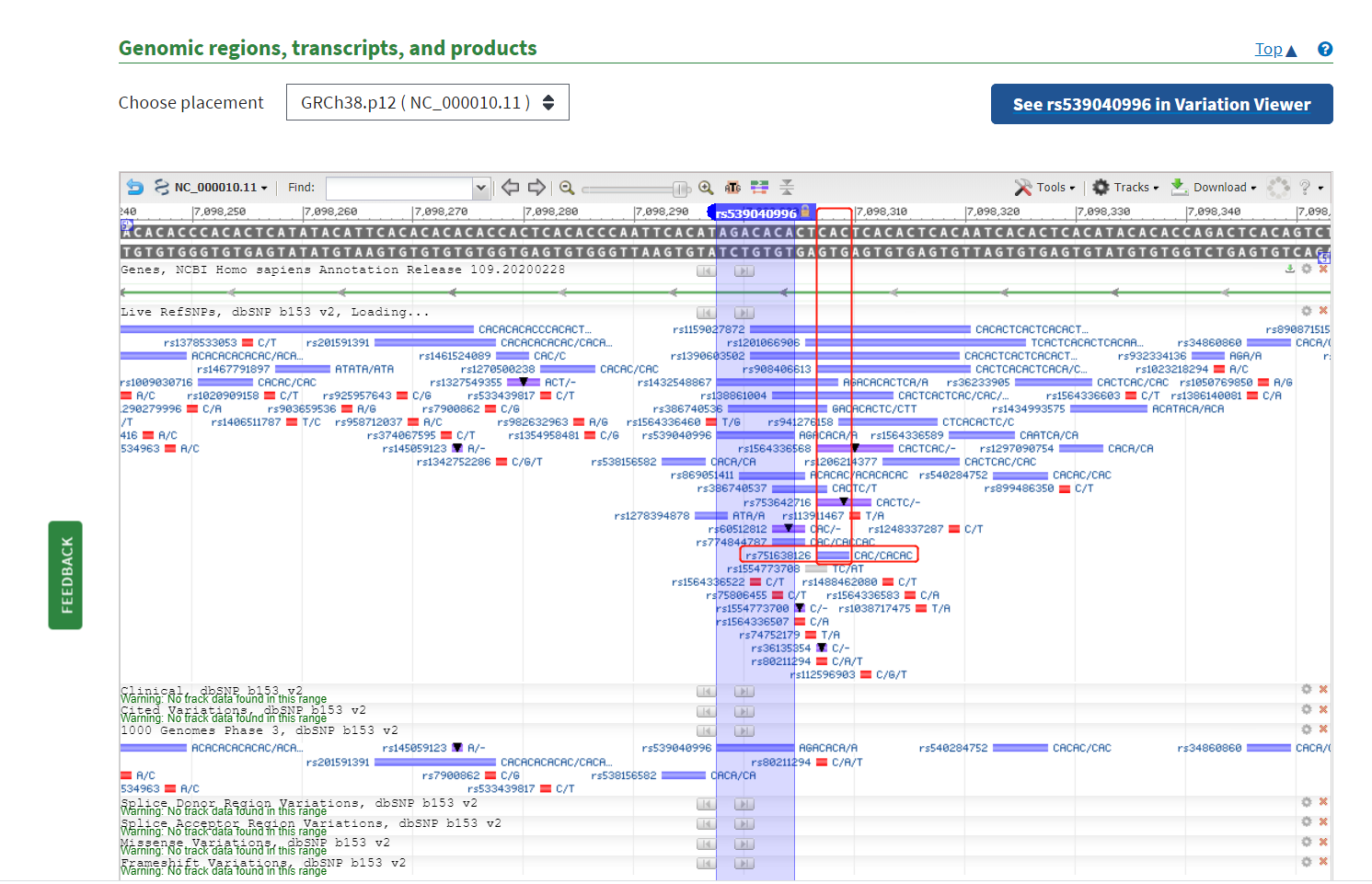
**
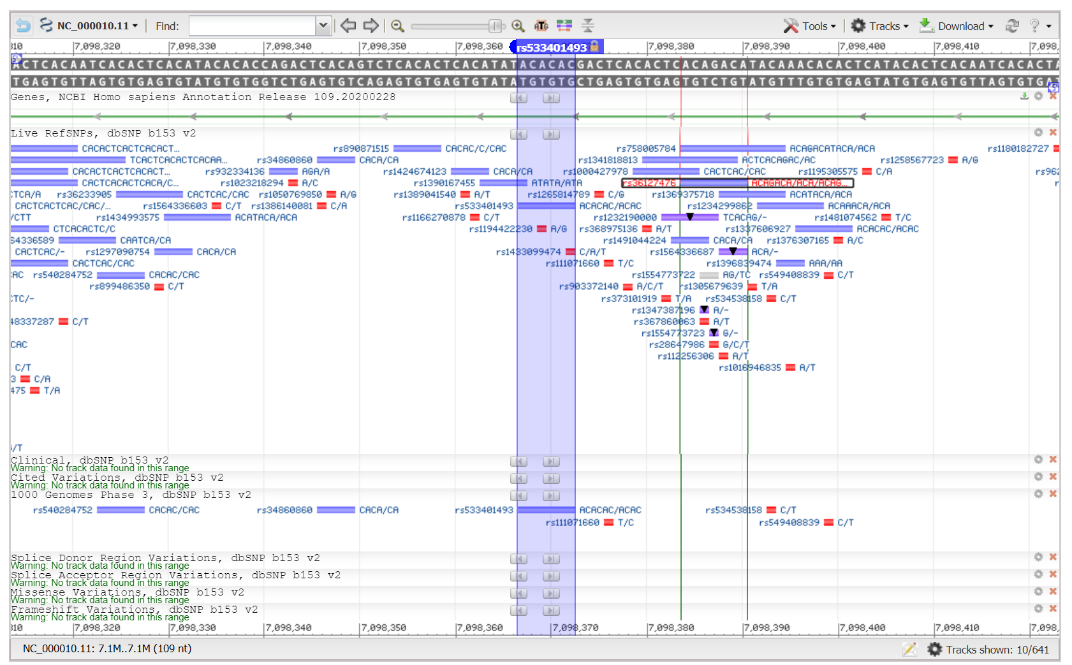


| **ID** | **Microhaplotype** | **GRCh37** | **rs-Number dbSNP** | **Extent in bp** | **Allele1/Allele2** | **Insertion allele length** | **Primer sequences (label)** | **The actual amplicon size (bp)** |
| --- | --- | --- | --- | --- | --- | --- | --- | --- |
| 40 | mh21zl002 | 42384738 | rs147144385 | 55 | T/TA | 1 | ACACATTCTCAAGCACTCACA (HEX) | 145 |
|  |  | 42384760 | rs200218606 |  | T/TCACA | 4 | GGTGGGAGATGTGAATGTGT |  |
|  |  | 42384793 | rs138205093 |  | A/ACT | 2 |  |  |


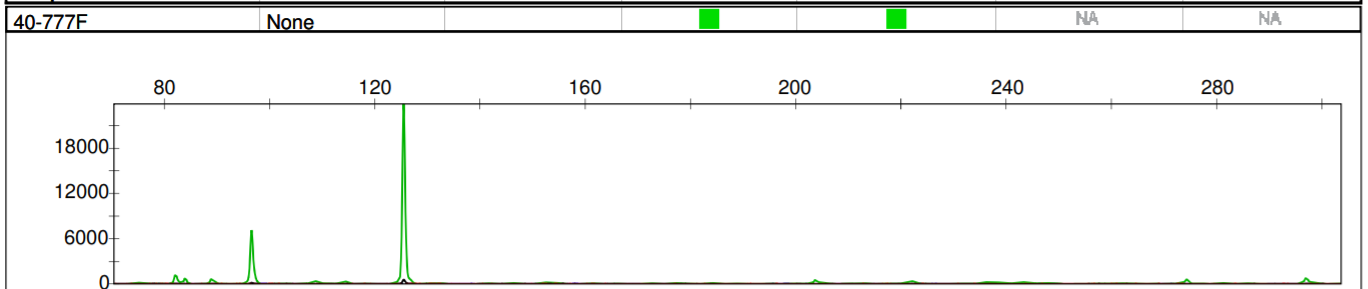


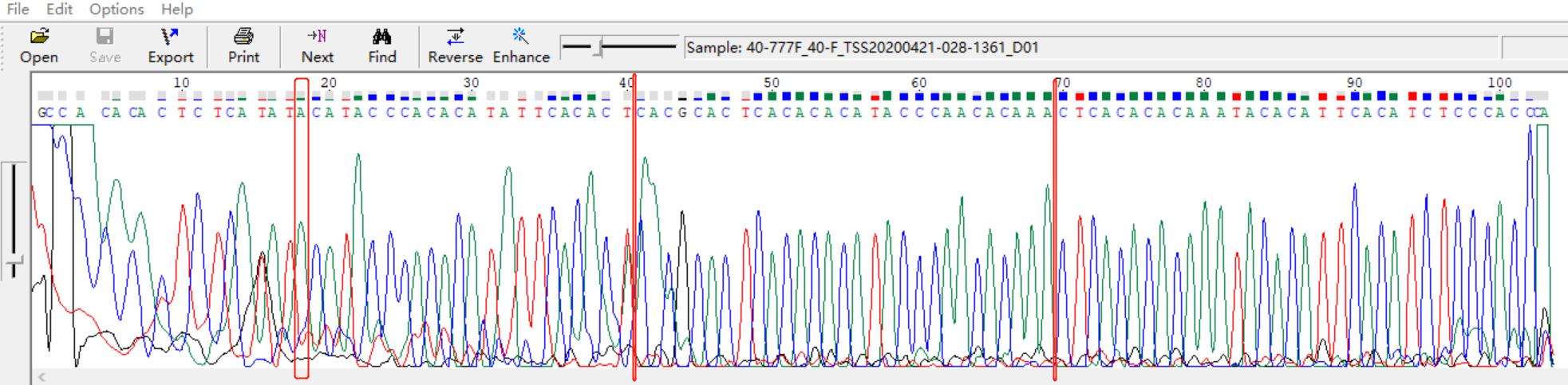


| **ID** | **Microhaplotype** | **GRCh37** | **rs-Number dbSNP** | **Extent in bp** | **Allele1/Allele2** | **Insertion allele length** | **Primer sequences (label)** | **The actual amplicon size (bp)** |
| --- | --- | --- | --- | --- | --- | --- | --- | --- |
| 42^*^ | mh02zl003 | 212161558 | rs575990766 | 85 | A/ACATATGTATG | 10 | ACTAAAGCCTGTATATGTAGCCT (ROX) | 166, 178, 226 |
|  |  | 212161604 | rs141442566 |  | A/ATACATATGTATGTATG | 16 | CCCAGTATCATTCTCTATCTCTGC |  |
|  |  | 212161643 | rs66617012 |  | A/ATAAG | 4 |  |  |

^*^The actual amplicon size at this locus is different from the theoretical amplicon size. Because there are some variations (rs1559083950 or others) that are not included in the 1000 Genomes Project phase 3. The sequencing results showed that the actual amplified fragment have more deletion variations. Therefore, the actual amplicon size is inconsistent with the theoretical amplicon size inferred from the 1000 Genomes Project phase 3.

Sample 1


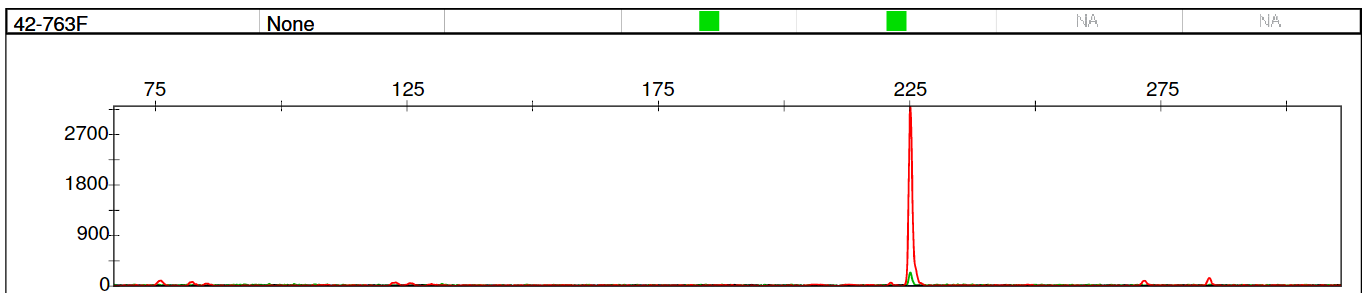


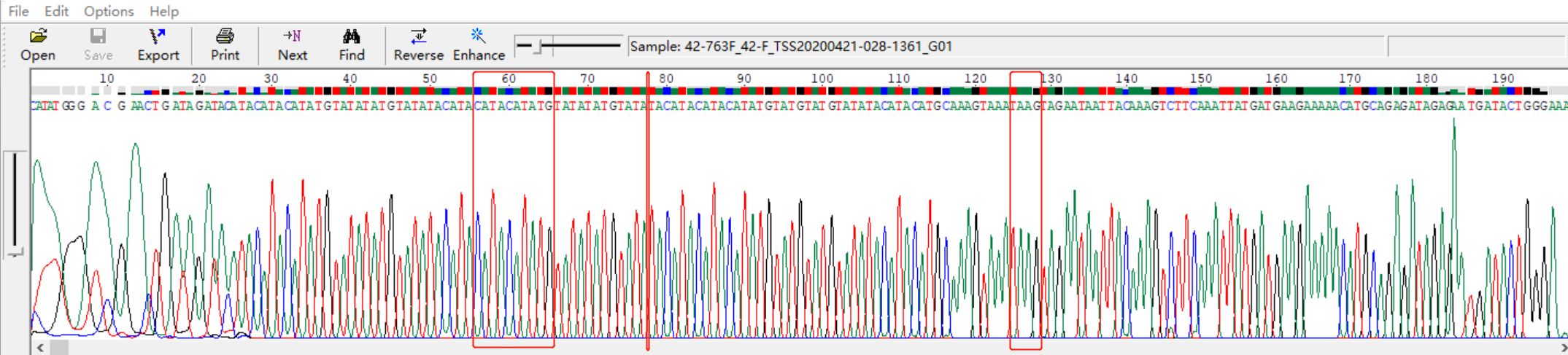


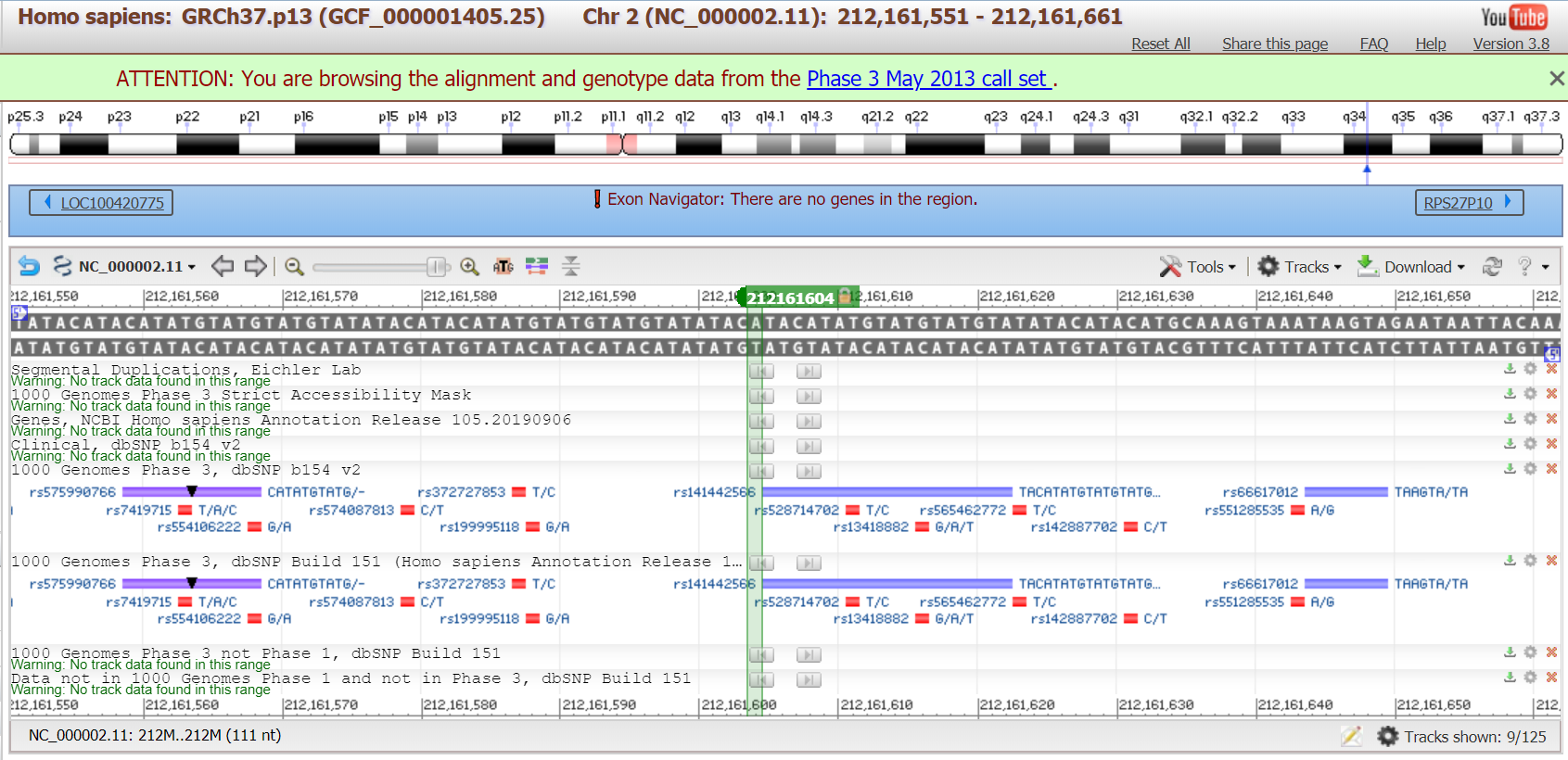

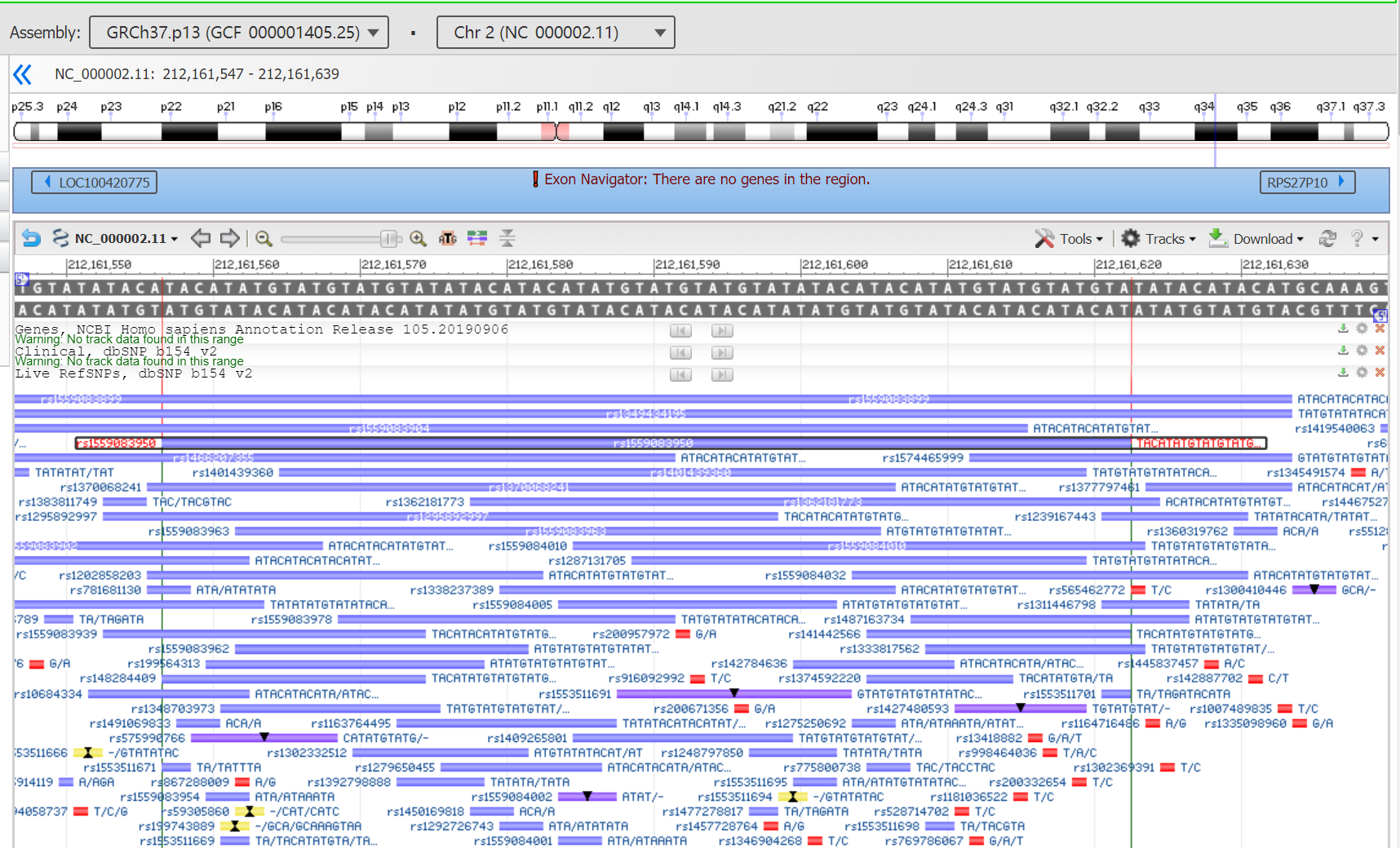


Sample 2


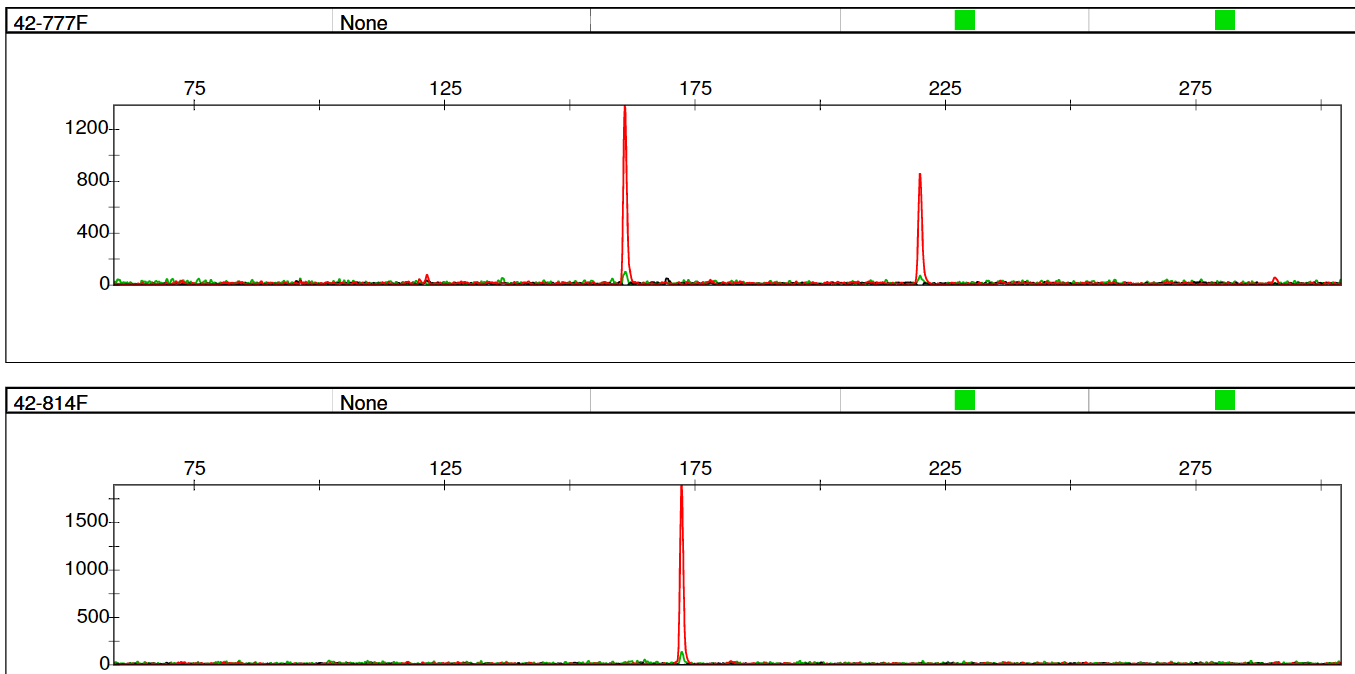


Allele 1


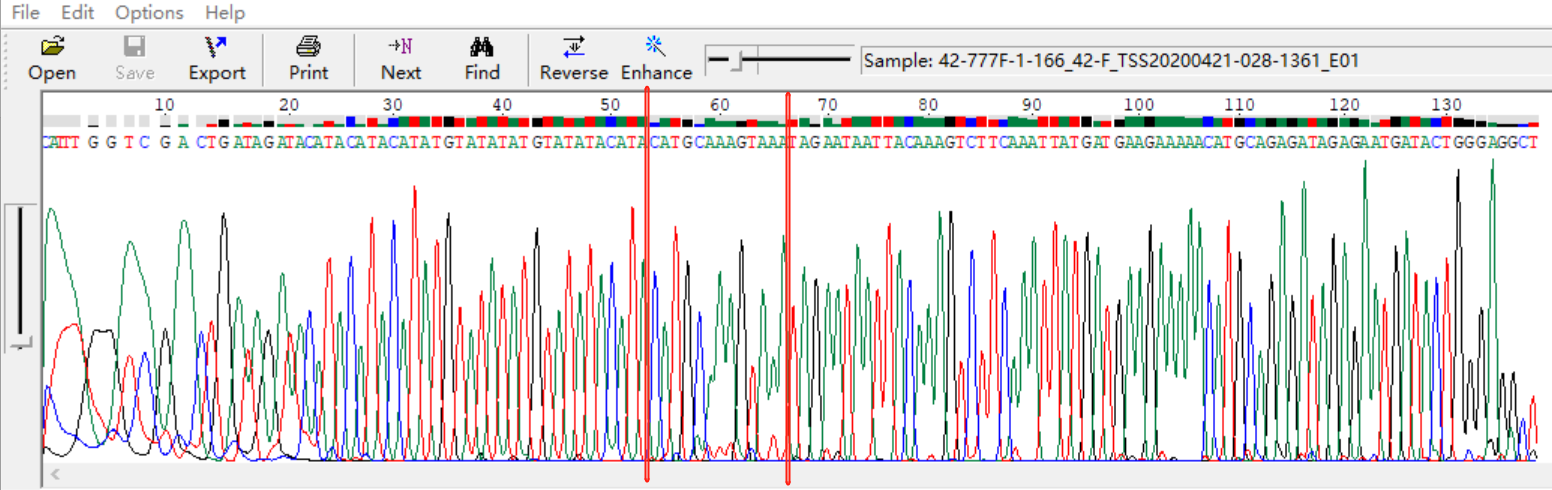


Allele 2


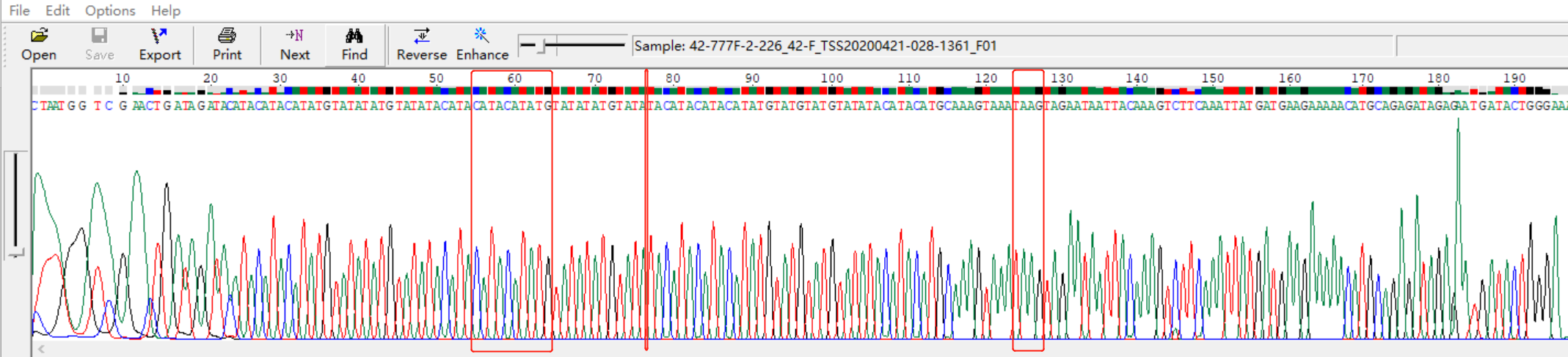


Sample 3


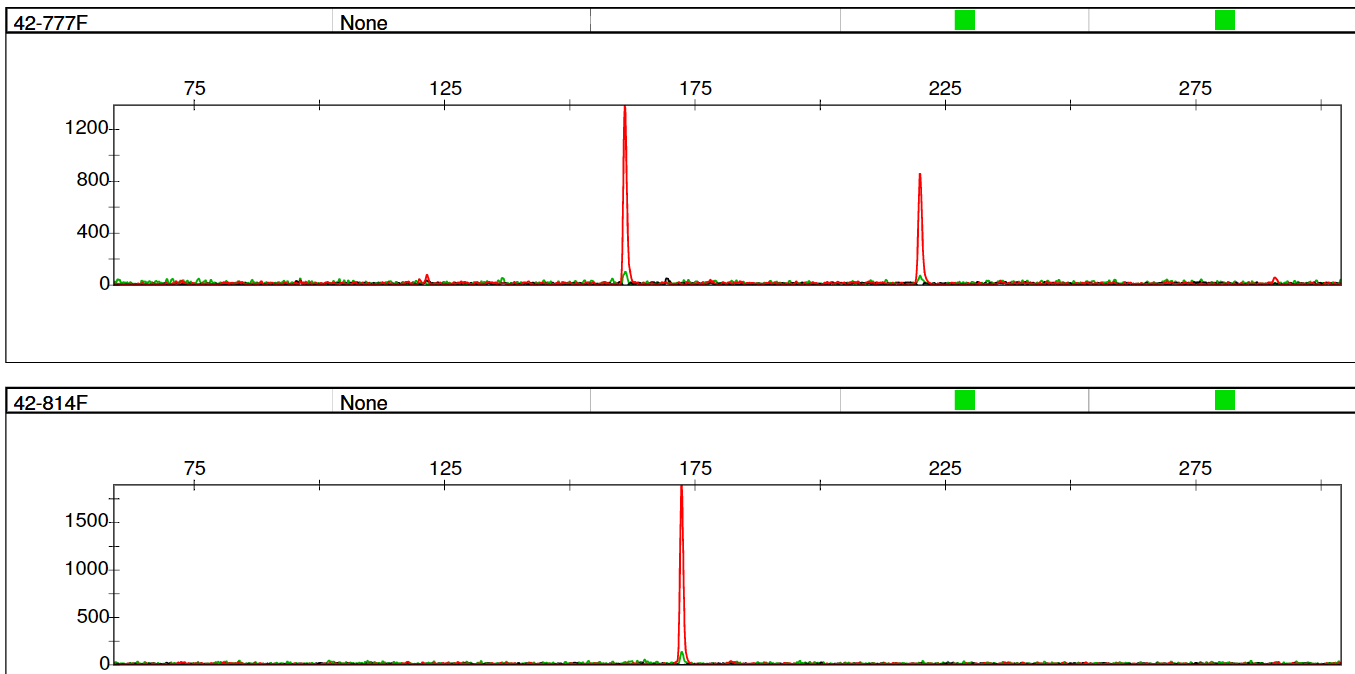


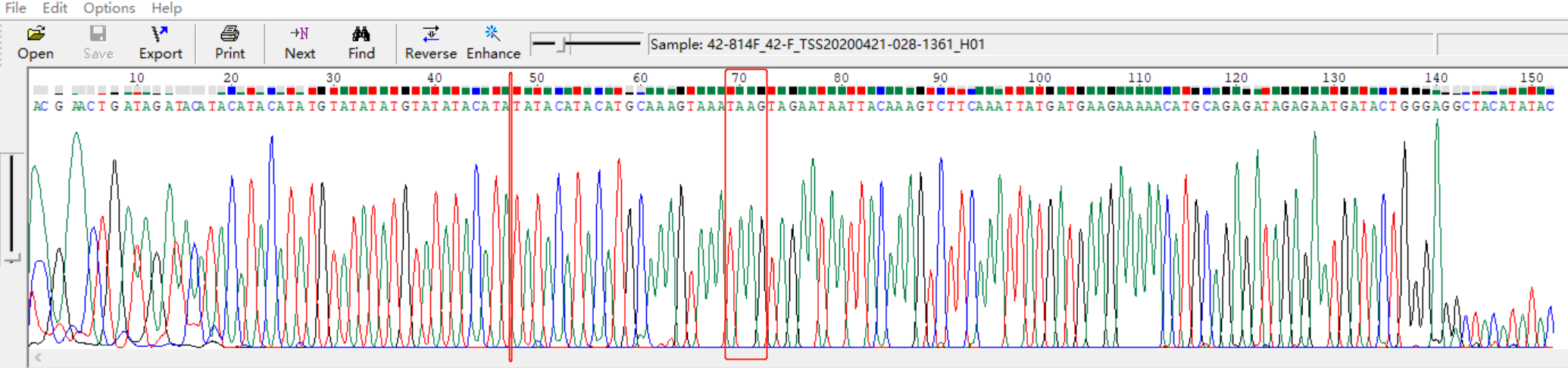

Supplement: Supplementary file 3 [file Table_1.DOCX]
